# Screening of Yeast Display Libraries of Enzymatically Treated Peptides to Discover Macrocyclic Peptide Ligands

**DOI:** 10.3390/ijms22041634

**Published:** 2021-02-05

**Authors:** John Bowen, John Schneible, Kaitlyn Bacon, Collin Labar, Stefano Menegatti, Balaji M. Rao

**Affiliations:** 1 Department of Chemical and Biomolecular Engineering, North Carolina State University, 911 Partners Way, Raleigh, NC 27695, USA; jdbowen3@ncsu.edu (J.B.); jdschnei@ncsu.edu (J.S.); kbbacon@ncsu.edu (K.B.); 2 Department of Molecular and Structural Biochemistry, North Carolina State University, Raleigh, NC 27695, USA; crlabar@ncsu.edu; 3 Biomanufacturing Training and Education Center (BTEC), North Carolina State University, 850 Oval Dr, Raleigh, NC 27606, USA

**Keywords:** yeast display library, cyclic peptide, Yes Associated Protein (YAP) 65, WW domain, transglutaminase

## Abstract

We present the construction and screening of yeast display libraries of post-translationally modified peptides wherein site-selective enzymatic treatment of linear peptides is achieved using bacterial transglutaminase. To this end, we developed two alternative routes, namely (i) yeast display of linear peptides followed by treatment with recombinant transglutaminase in solution; or (ii) intracellular co-expression of linear peptides and transglutaminase to achieve peptide modification in the endoplasmic reticulum prior to yeast surface display. The efficiency of peptide modification was evaluated via orthogonal detection of epitope tags integrated in the yeast-displayed peptides by flow cytometry, and via comparative cleavage of putative cyclic vs. linear peptides by tobacco etch virus (TEV) protease. Subsequently, yeast display libraries of transglutaminase-treated peptides were screened to isolate binders to the N-terminal region of the Yes-Associated Protein (YAP) and its WW domains using magnetic selection and fluorescence activated cell sorting (FACS). The identified peptide cyclo[*E-*LYLAYPAH*-K*] featured a K_D_ of 1.75 μM for YAP and 0.68 μM for the WW domains of YAP as well as high binding selectivity against albumin and lysozyme. These results demonstrate the usefulness of enzyme-mediated cyclization in screening combinatorial libraries to identify cyclic peptide binders.

## 1. Introduction

The use of cyclic peptides in basic research, as well as diagnostic and therapeutic applications, has grown significantly owing to their superior bioactivity and biochemical stability compared to their linear counterparts [1,2,3]. A particularly attractive feature of cyclic peptides is their small size, which enables their intercalation within protein-protein complexes resulting in site-selective inhibition [4,5]. Several platform technologies have been developed for the isolation of bioactive peptides via combinatorial screening, such as mRNA-display [6], phage-display [7], yeast-display [8] and solid-phase synthetic libraries [9]. Linear peptides with good target affinity can be easily developed using these methods [10,11,12]. However, not all peptide sequences selected in a linear form retain their biorecognition activity upon cyclization [13,14], and direct selection of peptides in their cyclic form from combinatorial libraries is therefore preferred.

Several strategies are available for generating combinatorial libraries of cyclized peptides. The formation of a disulfide bond between cysteine residues flanking the peptide sequence is the most widely employed to date [15,16,17]; disulfide-cyclized peptides, however, cannot be utilized in reducing environments, such as the intracellular fluid, which hydrolyze the disulfide linkage [18,19]. Therefore, cyclization methods that utilize reactive crosslinkers, such as disuccinimidyl glutarate [19,20,21] or iodoacetic anhydride [22], to form stable bonds have been developed. These crosslinkers, however, are promiscuous and can react with any residues displayed on the linear construct, resulting in unwanted crosslinking [22]. Enzyme-mediated ligation provides a site-selective route to achieve stable peptide cyclization, owing to the ability of enzymes to recognize and modify specific peptide motifs [23,24,25]. A wide range of enzymes have been employed to achieve peptide or protein cyclization such as sortase [26,27], butelase 1 [28], the S-adenosylmethionine enzyme AlbA [29], subtiligase [30], the thioesterase domain of tyrocidine synthetase [31], inteins [32,33,34,35], and transglutaminase [24]. Despite their proven potential, only a few examples are reported where enzymes were used to engineer display libraries of cyclic peptides for de novo discovery of bioactive peptides [36,37,38].

Here, we present a method for constructing yeast display libraries of cyclic peptides that are modified post-translationally by transglutaminase. This enzyme catalyzes the formation of an amide (i.e., peptide) bond between a glutamine (Q) residue preceded by a N-terminal ALQ tripeptide motif and a C-terminal lysine (K) residue, enabling peptide modifications such as “head-to-side chain”-type cyclization [24]; these amino acid sequence requirements must be consider when designing peptide libraries whose randomized sequences are generated using degenerate codons. Notably, transglutaminase can be recombinantly produced in *E. coli* in relatively high amounts and affordably, thus proving an ideal tool for the cyclization of cell-displayed peptides in vitro [39].

In our approach, a linear peptide precursor was displayed on the surface of yeast cells as a fusion to the Aga2p subunit of the yeast mating protein α-agglutinin [8]. The Aga2p subunit is linked to the Aga1p subunit of α-agglutinin via disulfide bonds that tether the linear peptide to the yeast cell surface. We attempted two routes to achieve transglutaminase-mediated putative cyclization of the linear peptide precursors. The first strategy comprised the expression of the linear peptide precursors on the yeast surface followed by the incubation of the cells with soluble recombinant transglutaminase. The second strategy relied on the concurrent intracellular expression and trafficking of the linear peptide precursors and transglutaminase, aimed at achieving cyclization of the peptides in the endoplasmic reticulum prior to their display on the yeast surface [40]. To evaluate the efficiency of transglutaminase-mediated cyclization, we developed a suite of indirect analytics that rely on the quantification of specific epitope tags framed within the peptide construct; as transglutaminase-mediated cyclization occurs, the immunofluorescent detection level of the tags decreases proportionally with the yield of peptide modification.

Using this toolbox, we evaluated the integration of transglutaminase-mediated cyclization with yeast display peptide to identify protein-targeting ligands. To this end, we combined magnetic selection and fluorescence activated cell sorting (FACS) in the screening of a yeast display combinatorial library of transglutaminase-modified peptides against the N-terminal region of the Yes-Associated Protein (YAP, AAs 1–291) and its WW domains (AAs 165–271). Since YAP plays a crucial role as a transcriptional regulator in cell proliferation and apoptosis, YAP-binding ligands can serve as modulators of the Hippo signaling pathway and provide control over cellular mechanisms responsible for organ and tumor development and growth [41]. Screening yielded several sequences, which were characterized via yeast surface titration and chromatographic binding assays as well as in silico docking simulations to assess their binding affinity and specificity to N-terminal YAP and WW-YAP. Selected peptide cyclo[*E*-LYLAYPAH-*K*] showed high affinity for N-terminal YAP and WW-YAP, thus demonstrating the feasibility of the proposed method as a platform for the de novo discovery of cyclic peptide binders.

## 2. Results and Discussion

### 2.1. Evaluation of Extracellular Transglutaminase-Mediated Putative Cyclization of Yeast-Displayed Peptides

**Figure 1:**
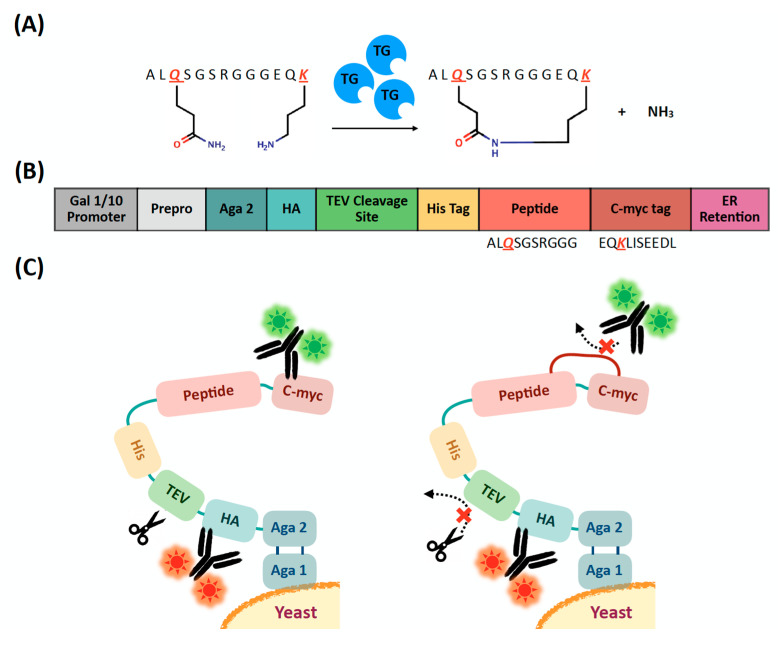
Transglutaminase-mediated (putative) cyclization of model peptides displayed on the surface of yeast cells and evaluation of the peptide cyclization efficiency. (**A**) Formation of an amide bond between the ε-amino group of a C-terminal lysine and the γ-carbamoyl side chain group of an N-terminal glutamine by transglutaminase; (**B**) Engineering of the pCTCON plasmid vector to express the model peptide sequence ALQSGSRGGG contiguously to the c-myc epitope sequence EQKLISEEDL; and (**C**) Schematics of peptide display on the yeast surface and impact of transglutaminase-mediate peptide (putative) cyclization on the detection of the c-myc and HA tags and the efficiency of cleavage by TEV protease.

Transglutaminase catalyzes the formation of an amide (i.e., peptide) bond between the ε-amino group of a C-terminal lysine and the γ-carbamoyl side chain group of an N-terminal glutamine (Figure 1A) and has been widely utilized for protein modification as well as peptide conjugation and cyclization [42]. In the context of cyclization of short peptides, transglutaminase has been utilized either alone or in combination with orthogonal cyclization agents to construct cyclic and polycyclic constructs comprising 6–12 residues [24,43]. Considerable efforts have been dedicated to the engineering of the enzyme [44], the identification of its recognition sequence, and the optimization of reaction conditions to increase the yield of ligation [24,45]; in particular, it has been recognized that appending the N-terminal dipeptide Ala-Leu significantly increases the cyclization yield [24].

In this study, we adopted the sequence ALQSGSRGGGKS as a model linear precursor to evaluate the efficiency of transglutaminase-mediated cyclization on the surface of yeast cells; this peptide, in fact, served as model substrate in prior work on transglutaminase-mediated cyclization [24]. Specifically, we designed a plasmid construct to express the lead sequence ALQSGSRGGG as an Aga2p fusion on the surface of *Saccharomyces cerevisiae* cells. Together with the model peptide, the Aga2p fusion construct contains c-myc and HA epitope tags as well as a tobacco etch virus (TEV) protease signal recognition sequence (Figure 1B). Notably, the c-myc epitope, EQKLISEEDL, provides the lysine residue (K) to be enzymatically ligated with the glutamine (Q) of the lead sequence, ALQSGSRGGG, affording (putative) peptide cyclization. We hypothesized that transglutaminase-mediated tethering of the lead sequence and the c-myc tag hinders—or completely prevents—the binding of c-myc antibodies (Figure 1C). Accordingly, quantification of the differential binding of fluorescently labeled c-myc antibodies to the peptide constructs displayed on cells that were either treated transglutaminase or left untreated provides an assessment of the putative cyclization (note: to ensure the site-selectivity of the cyclization, we eliminated the lysine and serine residues from the C-terminus of the model peptide sequence).

We initially attempted to quantify on-yeast cyclization by high-resolution mass spectrometry. We analyzed Aga2p-peptide fusions cleaved from the cell surface by treatment with a reducing agent. However, due to the low abundance and high complexity of the cleavage products, we were unable to differentiate unambiguously between linear and cyclic peptides. Therefore, we developed an indirect method to assess peptide cyclization, based on enzymatic treatment and fluorescence detection of specially-located epitopes.

**Figure 2:**
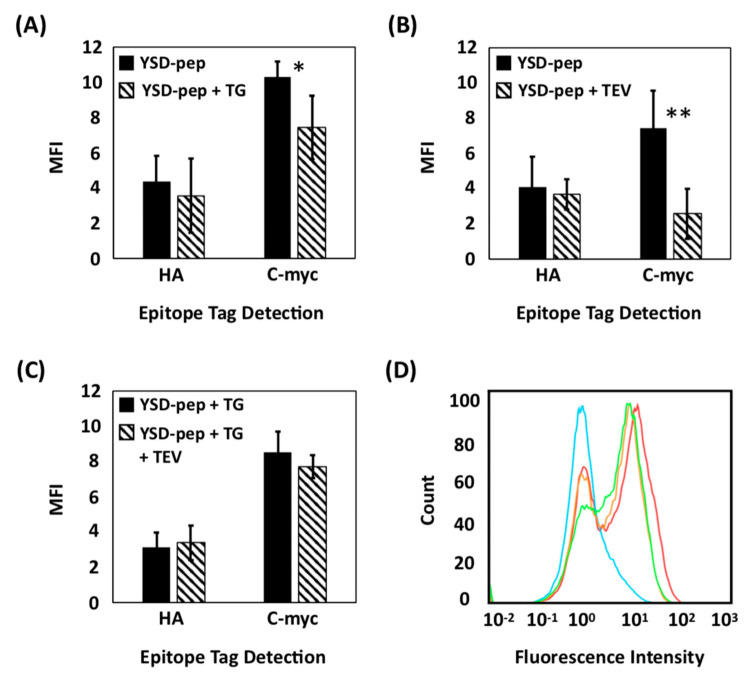
Evaluation of the yield of transglutaminase-mediated (putative) cyclization of yeast displayed peptides via immunofluorescence labeling and flow cytometry. (**A**) Mean fluorescence intensity (MFI) of HA and c-myc tag levels detected on yeast cells expressing a linear peptide sequence that were either not treated (YSD-Pep) or treated with transglutaminase (YSD-pep + TG); (**B**) MFI of HA and c-myc tag levels detected on yeast cells expressing a linear peptide sequence that were either not treated (YSD-pep) or treated with TEV protease (YSD-Pep + TEV). (**C**) MFI of HA and c-myc tag levels detected on yeast cells expressing a linear peptide sequence that were either treated with transglutaminase only (YSD-pep + TG) or transglutaminase and TEV protease (YSD-pep + TG + TEV); and (**D**) Representative flow cytometry histograms of c-myc epitope levels detected on yeast cells expressing a linear peptide sequence that received either no treatment (red), treatment with transglutaminase only (orange), treatment with TEV protease only (cyan), or treatment with transglutaminase and TEV protease (green). The error bars correspond to the standard error of the mean from three independent replicates. A two-tailed paired t-test was performed; * indicates *p* < 0.1 and ** indicates *p* < 0.05.

After induction of linear peptide display, cells were either treated with soluble recombinant transglutaminase or plain buffer (negative control). The expression of the peptide was confirmed by detecting the HA and c-myc tags via flow cytometry (Figure 2A,D). While the HA signal did not differ between transglutaminase-treated and untreated cells, a notable decrease in the c-myc signal was observed for cells incubated with transglutaminase. This is consistent with our hypothesis of decreased antibody binding to the c-myc tag upon enzymatic crosslinking of the model peptide sequence.

We further evaluated the alleged cyclization yield by assessing the extent of cleavage of transglutaminase-treated vs. untreated peptide constructs from yeast cells by TEV protease. The latter recognizes the sequence ENLYFQ-G/S and cleaves between the Q and G/S residues [40]. We hypothesize that peptide modifications—such as cyclization –inhibit TEV protease from interacting with its recognition sequence included between the construct’s His tag and HA tag. Accordingly, yeast cells expressing the linear peptide precursor—that were either treated with transglutaminase or left untreated—were incubated with TEV protease, and the binding of antibodies targeting the HA and c-myc tags was quantified via flow cytometry. No variation in HA signal upon treatment with TEV protease was observed, irrespective of transglutaminase treatment (Figure 2B,C); this was expected, given that the TEV recognition sequence is in downstream of the HA tag (Figure 1C). However, a notable difference in residual c-myc level was observed between untreated cells, which respectively showed a significant decrease in fluorescence (Figure 2B,D), and cells treated with transglutaminase, which showed only a slight decrease (Figure 2C,D), after TEV treatment. This is coherent with our hypothesis that the TEV recognition site is significantly sterically hindered upon the alleged peptide cyclization or is in itself involved in the putative cyclization through its glutamine residue (*note:* the latter is unlikely due to the lack of Ala-Leu leader sequence required for transglutaminase reaction within the TEV recognition site). Collectively, these results suggest that extracellular treatment with transglutaminase results in the (putative) cyclization of the linear peptide constructs expressed as yeast surface fusions.

### 2.2. Design, Construction, and Screening of a Yeast Display Library of Peptides Modified Using Extracellular Transglutaminase

**Figure 3:**
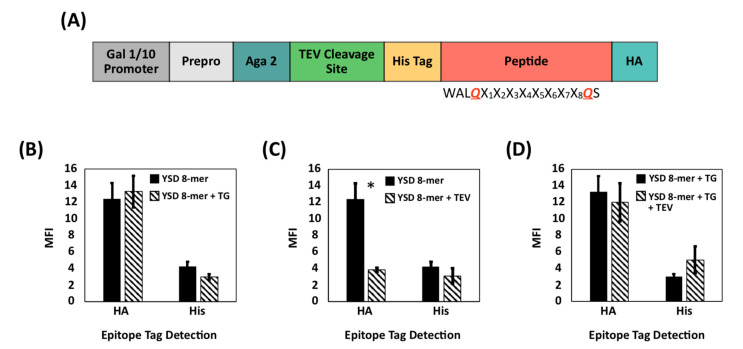
Evaluation of the yield of transglutaminase-mediated (putative) cyclization of a yeast displayed peptide library via immunofluorescence labeling and flow cytometry. (**A**) Engineering of the pCTCON plasmid to express a combinatorial library of 8-mer peptides as yeast surface fusions; (**B**) MFI of HA and His tag levels detected on the cells from a yeast display library of 8-mer peptides that were either not treated (YSD 8-mer) or treated with transglutaminase (YSD 8-mer + TG); (**C**) MFI of HA and His tag levels detected on the cells from a yeast surface display library of 8-mer peptides that were either not treated (YSD 8-mer) or treated with TEV protease only (YSD 8-mer + TEV); and (**D**) MFI of HA and His tag levels detected on the cells from a yeast surface display library of 8-mer peptides that were either treated with transglutaminase only (YSD 8-mer + TG) or transglutaminase and TEV protease (YSD 8-mer + TG + TEV). Error bars correspond to the standard error of the mean from three independent replicates. A two-tailed paired t-test was performed. * *p* < 0.05.

We utilized the method of extracellular peptide cyclization demonstrated above to construct a yeast display library of cyclic peptides. To this end, an 8-mer randomized linear peptide sequence, WALQX_1_ X_2_-X_3_-X_4_-X_5_-X_6_-X_7_-X_8_KS, was expressed and modified by extracellular transglutaminase (Figure 3A, note: because a C-terminal lysine (K) was incorporated in the library sequence, the c-myc tag was omitted from the construct). The modification efficiency of transglutaminase on the linear peptide precursors displayed on the library cells was evaluated using the indirect fluorescent labeling method described. Specifically, we evaluated both the binding of antibodies to the HA and His tags framed within the library peptide-Aga2p fusions and the reduction of peptide cleavage by the TEV protease following treatment with transglutaminase. As anticipated, the antibodies targeting the HA tag bound similarly to transglutaminase-treated and untreated library cells (Figure 3B); exposure to TEV protease resulted in a notable decrease in the binding of antibodies targeting the His tag on the untreated yeast display library (Figure 3C), whereas no significant variation was observed for the yeast display library of transglutaminase-treated peptides (Figure 3D). These results suggest that an effective modification—such as cyclization—of the peptide yeast library was achieved with transglutaminase.

We then screened the library against the N-terminal region of Yes-Associated Protein 65 (N-YAP) and its WW domains (WW-YAP) using two rounds of magnetic selection followed by two rounds of FACS. Ten individual clones were sequenced for identification. Notably, 9 out of the 10 clones obtained from the screening against N-YAP and all the 10 clones obtained from the screening against WW-YAP returned the peptide sequence QLYLAYPAHK. This corresponds to the alleged cyclic peptide construct cyclo[*E*-LYLAYPAH-*K*], since the transglutaminase-mediated transamidation of the carbamoylethyl side chain group of glutamine (Q) with the ε-amine group of lysine (K) is equivalent to the formal amidation of the carboxyethyl side chain group of a glutamic acid (E) by the ε-amine group of lysine (K).

### 2.3. Evaluation of Binding Affinity and Specificity of (Putative) Cyclo[E-LYLAYPAH-K] via Yeast Surface Titrations

**Figure 4:**
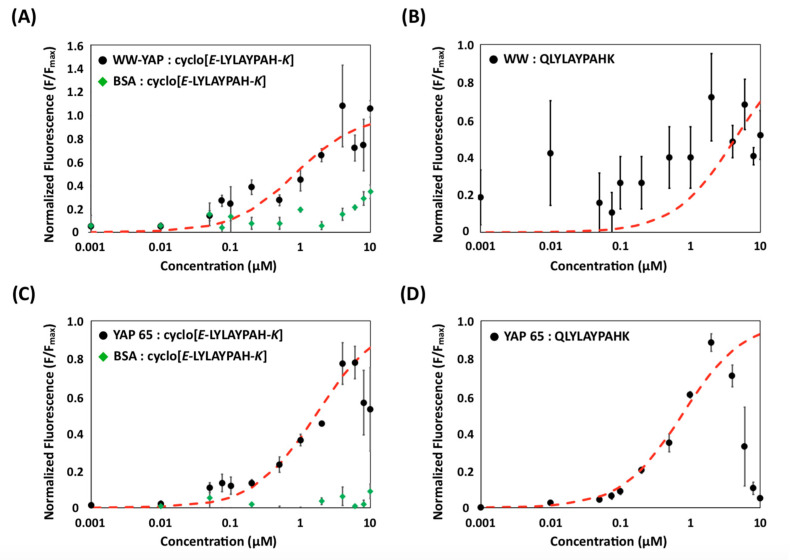
Surface titrations of (**A**) yeast cells displaying (putative) cyclo[*E*-LYLAYPAH-*K*] with the WW domain of YAP (WW-YAP, black circles) and bovine serum albumin (BSA, green diamonds); (**B**) yeast cells displaying the linear peptide sequence QLYLAYPAHK with WW-YAP; (**C**) yeast cells displaying (putative) cyclo[*E*-LYLAYPAH-*K*] with N-terminal-YAP (N-YAP, black circles) and BSA (green diamonds); and (**D**) yeast cells displaying the linear peptide sequence QLYLAYPAHK with N-YAP. The binding of each biotinylated protein was detected using SA-PE followed by flow cytometry analysis. Data from each of the three independent replicates were normalized to its associated F_max_ value. The resulting values of normalized mean fluorescence intensity for each repeat were fit against a monovalent binding isotherm (red dashed line) to estimate the apparent K_D_ describing the binding affinity of the yeast displayed peptides for the soluble protein targets. Error bars correspond to the standard error of the mean from three independent replicates.

The binding affinity of cyclo[*E*-LYLAYPAH-*K*] for N-terminal YAP and WW-YAP was characterized using a yeast surface titration method developed in prior work [21]. Specifically, yeast cells displaying (putative) cyclo[*E*-LYLAYPAH-*K*] were incubated with soluble, biotinylated N-terminal YAP or WW-YAP to generate binding isotherms [21]; the fraction of cell surface fusions bound by the biotinylated target protein was quantified via immunofluorescent detection of SA-PE (Figure 4). The resulting data were fit to a monovalent binding isotherm to estimate the binding affinity (*K_D_*) of the peptide (Table 1). Binding isotherms were also generated using BSA (Figure 4A,C), to characterize the binding selectivity, as well as for cells displaying the linear precursor (QLYLAYPAHK), to evaluate the effect of cyclization on the binding activity of the identified peptide sequence (Figure 4B,D).

Most notably, the cells treated with transglutaminase exhibited a significantly higher fluorescence when titrated with WW-YAP than BSA (Figure 4A). Cells displaying (putative) cyclo[*E*-LYLAYPAH-*K*] returned an apparent K_D_ of 0.68 ± 0.21 μM, which is consistent with other WW domain binding peptides [46]; the corresponding value for BSA could not be calculated, as the isotherm did not reach saturation within the tested protein concentration range. Furthermore, the linear precursors exhibited a much lower binding affinity (Figure 4B), with no obvious isotherm-like binding behavior. This indicates that transglutaminase-mediated treatment endows the peptide with specific and strong WW-binding activity.

Similarly, the apparent K_D_ of (putative) cyclo[*E*-LYLAYPAH-*K*] to N-terminal YAP was 1.75 ± 0.33 μM (Figure 4C). The peptide was found to bind N-terminal YAP specifically, as it exhibited little-to-no BSA binding. Notably, the linear form of the peptide was shown to bind to N-terminal YAP, but not WW-YAP (Figure 4D). We note that, without having performed any selection to ensure that the linear peptide does not interact with the target protein, it is possible that the linear precursor targets a region of N-terminal YAP outside of its WW domains.

### 2.4. Evaluation of Binding Affinity and Specificity of Cyclo[E-LYLAYPAH-K] via Static Binding Assays on Chromatographic Resins

**Figure 5:**
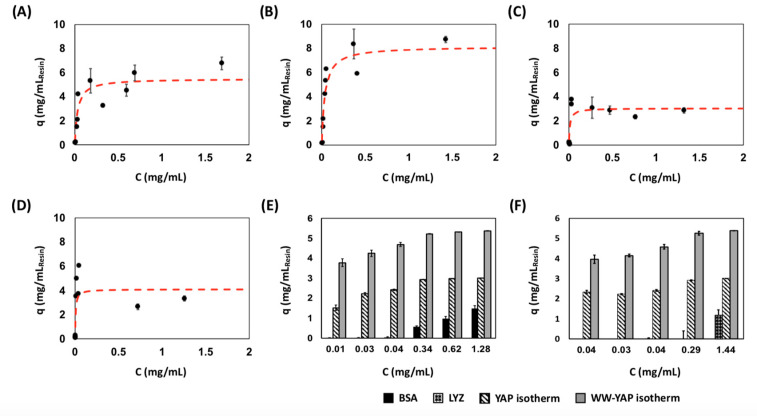
Isotherm binding values of (**A**) WW-YAP on RYSPPPPYSSHS-Toyopearl resin; (**B**) WW-YAP on cyclo[*E*-LYLAYPAH-*K*]-Toyopearl resin; (**C**) N-terminal YAP (N-YAP) on RYSPPPPYSSHS-Toyopearl resin; (**D**) N-YAP on cyclo[*E*-LYLAYPAH-*K*]-Toyopearl resin; (**E**) Comparison of bovine serum albumin (BSA) adsorption (mg protein/mL resin) to WW and N-YAP adsorption at similar values of equilibrium concentration; and (**F**) Comparison of lysozyme (LYZ), WW, and N-terminal YAP adsorption (mg protein/mL resin) at similar equilibrium protein concentrations. Error bars correspond to the standard error of the mean from three independent replicates. The amounts of WW-YAP and N-terminal YAP adsorbed were statistically different from those of the non-target proteins (BSA or lysozyme) at every value of equilibrium concentration, as demonstrated using a two-tailed unequal variance t-test (*p* < 0.05).

The binding analysis performed via yeast surface titrations suggested that (putative) cyclo[E-LYLAYPAH-K] exhibits selective binding to WW-YAP and bound with higher affinity than its linear counterpart. We further evaluated the biorecognition activity of cyclo[*E*-LYLAYPAH-*K*] as a synthetic construct on solid phase. To this end, the peptide sequence, where an N-terminal glutamic acid (E) was used instead of glutamine (Q), was synthesized on Toyopearl amino resin and cyclized by reacting the γ-carboxylic acid of E with the ε-amino group of K. This tethering strategy makes the cyclic synthesized peptide chemically identical to that obtained by transglutaminase-mediated cyclization. The known WW-binding peptide sequence RYSPPPPYSSHS (PTCH peptide) was also conjugated on Toyopearl resin to serve as positive control. Protein adsorption isotherms were generated by incubating the peptide-functionalized resin with soluble N-terminal YAP or WW-YAP (20 μg/mL–3 mg/mL). The adsorption data (i.e., the amounts of protein adsorbed on solid phase vs. the corresponding values of protein concentration in solution at the binding equilibrium) of WW-YAP on PTCH-Toyopearl and cyclo[*E*-LYLAYPAH-*K*]-Toyopearl resins are reported in Figure 5A,B, respectively; the corresponding binding isotherms for N-terminal YAP are presented Figure 5C,D, respectively.

The values of K_D_ and Q_max_ obtained by fitting the adsorption data to a Langmuir binding isotherm are collated in Table 2. Notably, the values of apparent K_D_ of cyclo[*E*-LYLAYPAH-*K*] are an order of magnitude lower (i.e., higher binding strength) than those estimated from yeast surface titrations. This can be imputed to the higher surface density of peptide ligands conjugated on Toyopearl resin (~7 μmol/m^2^) compared to the surface density of peptides displayed on the yeast surface (~2 nmol/m^2^). It is also important to note that, while the adsorption isotherms describing the binding of WW-YAP to the resins functionalized with the cyclic peptide or the PTCH peptide feature a standard monovalent binding isotherm shape, those obtained with N-terminal YAP are rather irregular. Specifically, both isotherms display the characteristic rapid increase in soluble protein binding within the range of equilibrium protein concentration below 0.5 mg/mL; however, as the latter approaches and exceeds~1 mg/mL, the isotherms do not feature the characteristic plateau, but exhibit a slight decrease in the amount of adsorbed protein. This suggests that the layer of protein adsorbed on the surface of peptide-functionalized resin may be unstable, and the free YAP in solution may interfere with the YAP:peptide complexes and trigger their dissociation.

We also evaluated the binding specificity of resin-bound cyclo[*E*-LYLAYPAH-*K*] using BSA and lysozyme (LYZ) as negatively and positively charged control proteins, respectively. The resulting adsorption data were compared to the adsorption data of WW-YAP and N-terminal YAP at similar equilibrium protein concentrations. These comparisons, summarized in Figure 5E for BSA and Figure 5F for LYZ, show significantly lower binding of both control proteins, demonstrating the specific biorecognition activity of cyclo[*E*-LYLAYPAH-*K*] for WW-YAP and N-terminal YAP.

Collectively, these results demonstrate that cyclic peptide binders with specific biorecognition activity and selectivity can be isolated from transglutaminase-treated yeast display peptide libraries.

### 2.5. Evaluation of Peptide Binding via Molecular Docking and Dynamics Simulations

To confirm the values of affinity obtained from the yeast surface titration and on-resin binding assays, we evaluated the binding strength of peptide cyclo[*E*-LYLAYPAH-*K*]-GSG and its linear precursor QLYLAYPAHK in silico via molecular docking and dynamics simulations; the interaction of the WW-binding Smad7 peptide (GESPPPPYSRYPMD) was also simulated as a positive control. The structure of the peptides was initially generated by molecular dynamics (MD) and subsequently docked against WW1-YAP (PDB ID: 2LTW) and WW2-YAP (PDB ID: 2LTV) [47] using HADDOCK (High Ambiguity Driven Protein-Protein Docking) [48]. The docked WW:peptide complexes were ranked, and selected binding poses were refined via MD to evaluate the free energy of binding (ΔG_b_) and the corresponding values of affinity (K_D_).

**Figure 6:**
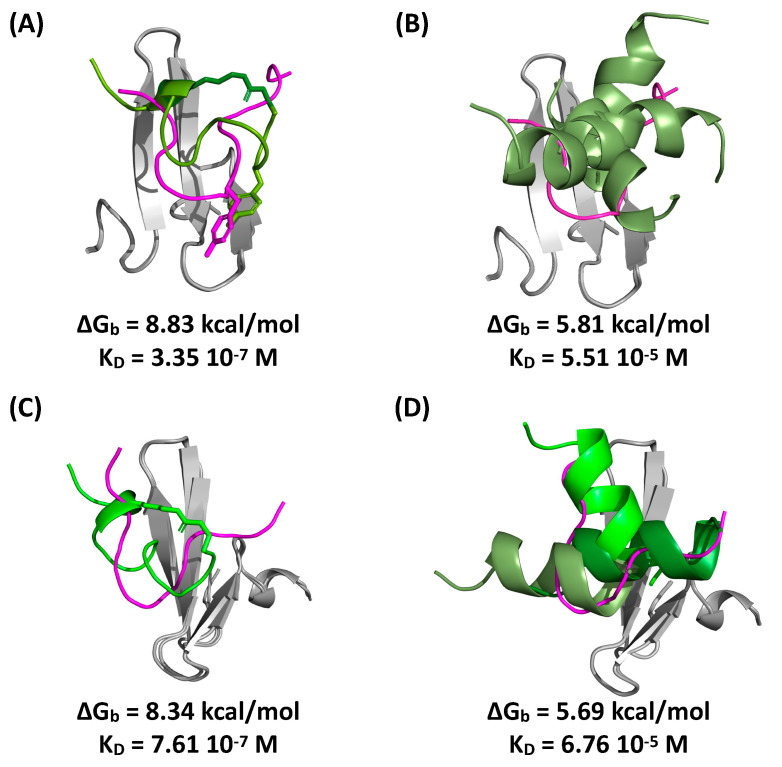
In silico complexes of WW1-YAP with (**A**) cyclo[*E*-LYLAYPAH-*K*]-GSG and (**B**) QLYLAYPAHK, and WW2-YAP with (**C**) cyclo[*E*-LYLAYPAH-*K*]-GSG and (**D**) QLYLAYPAHK obtained by molecular docking and dynamics simulations; the superimposition of Smad7 peptide (from PDB IDs 2LTW and 2LTV) with the selected WW:peptide complexes shows the overlapping binding epitopes of the identified peptides and Smad7 to the WW domains. WW1-YAP is in grey, cyclo[*E*-LYLAYPAH-*K*]-GSG and QLYLAYPAHK are in green, and the Smad7 peptide is in magenta. Values of binding energy (ΔG_b_) and corresponding affinity (K_D_) of the WW:peptide complexes are noted.

Representative poses of the peptides on WW1 and WW2 are reported in Figure 6 together with the corresponding values of K_D_. These results suggest that cyclo[*E*-LYLAYPAH-*K*]-GSG targets WW1 and WW2 in a similar region as the Smad7 peptide. Moreover, the affinity of cyclo[*E*-LYLAYPAH-*K*]-GSG to WW1 and WW2 is substantially higher than that of its linear counterpart. This finding is in agreement with the binding affinities predicted via yeast surface titration obtained with soluble WW domain. However, given the likelihood that the WW domains alone exhibit a conformation that is substantially similar to that of the WW domains within N-terminal YAP [49], a similar binding of the N-terminal YAP and the WW domains would be expected by both cyclo[*E*-LYLAYPAH-*K*] and its linear precursor. Thus, to justify the differences observed in silico and in the yeast surface titrations, we reason that the linear precursor QLYLAYPAHK likely binds N-terminal YAP (also) outside of its WW domains.

### 2.6. Evaluation of Peptide Cyclization in Yeast Cells by Intracellular Transglutaminase

While transglutaminase can be produced recombinantly in large quantities, significant processing is required to purify the enzyme from the recombinant source fluids. To circumvent this process, we explored the approach of engineering the yeast cells that express the linear peptides to concurrently produce transglutaminase (Figure S1). We anticipate that transglutaminase, while transported through the endoplasmic reticulum, will cyclize the linear peptides prior to their display on the yeast surface. To this end, we relied on the features of the pCTCON yeast surface display plasmid—specifically its bidirectional Gal1/Gal10 promoter—to express the linear peptide-Aga2 fusion under the control of the Gal1 promoter and transglutaminase under the control of the Gal10 promoter (Figure S2A). The model peptide sequence AL**Q**SGSRGGG-EQ**K**LISEEDL was adopted to evaluate the efficiency of cyclization by transglutaminase expressed intracellularly using the immunofluorescent epitope detection method.

Our initial construct expressed the full-length transglutaminase, which includes a pro-peptide sequence that is typically cleaved after translation. When expressing full-length transglutaminase, however, the epitope detection assays suggested that the displayed peptides were not modified (Figure S2B–E). Transglutaminase’s pro-peptide sequence forms α-helix that may occlude its active site. We therefore hypothesized that the pro-peptide sequence of transglutaminase was not cleaved and blocked the enzyme’s active site, thus preventing cyclization of the model peptide sequence.

To overcome this issue, we generated two additional constructs that presumably express transglutaminase in its active form (Figure S3). One variant introduced a Kex2 endopeptidase cleavage site between the pro-peptide and the active transglutaminase sequence. As Kex2 peptidase is endogenously expressed in yeast, we hypothesized that endogenous Kex2 peptidase will cleave the pro-peptide sequence resulting in an active form of transglutaminase. However, the insertion of the Kex2 recognition site did not improve the cyclization efficiency as shown by the epitope detection assays (Figures S4A,B and S5A,B. In our second variant, we expressed only the active sequence of transglutaminase (AA 61–395) by removing the pro-peptide sequence completely. When evaluating this construct, the epitope detection assays suggested that the linear peptide sequences had undergone partial cyclization (Figures S4C,D and S5C,D); additional details on the optimization of intracellular transglutaminase expression are provided in the Supplementary Materials.

Upon optimization of intracellular transglutaminase expression, we constructed a yeast display library that concurrently expressed the active form of transglutaminase and a linear peptide sequence containing seven randomized positions. The library was screened to identify cyclic peptide binders with affinity for N-terminal YAP. Following one round of magnetic selection and one round of FACS, the library was enriched for binding to N-terminal YAP (Figure S6). Sequencing of the selected clones returned one mutant, cyclo[*E*-VQCRGKGEQ-*K*], which was evaluated for its affinity and specificity to N-terminal YAP. The binding affinity of cyclo[*E*-VQCRGKGEQ-*K*] for N-terminal YAP was estimated as 0.46 ± 0.08 μM using the yeast surface titration method described (Figure S7A); the parental linear peptide QVQCRGKGEQK, however, showed a similar affinity (0.36 ± 0.07 μM, Figure S7B). The apparent similarity in N-terminal YAP binding to yeast cells displaying the linear and putative cyclic form of the peptide may be attributed to incomplete cyclization of the displayed peptides by the transglutaminase expressed intracellularly. The selection of yeast cells displaying a combination of cyclic and linear peptides may in fact lead to the identification of peptide sequences that bind the target protein in both configurations with comparable affinity. Additional details on the library selection process and characterization of peptides isolated from the yeast display library concurrently expressing intracellular transglutaminase are provided in the Supplementary Materials. Further optimization is needed to increase the expression of active transglutaminase within yeast cells to improve the cyclization efficiency and thereby increase the proportion of displayed-peptides that are cyclized on the yeast surface.

## 3. Materials and Methods

### 3.1. Plasmids and Yeast Cell Culture

The pCTCON vector containing the TRP selectable marker was used in conjugation with *Saccharomyces cerevisiae* strain EBY100. The Frozen-EZ yeast transformation Kit II (Zymo Research, Irvine, CA, USA) was used to transform plasmid DNA into chemically competent EBY100. Trp-deficient SDCAA and SGCAA media were used for culturing and inducing protein expression, respectively, as previously described [50]. During routine culture, yeast cells were incubated at 30 °C while shaking at 250 RPM. To induce protein expression, yeast cells were transferred into SGCAA media at OD_600_ of 1 and incubated for 24 h or 48 h at 20 °C while shaking at 250 rpm. EBY100 yeast without plasmid was grown in YPD medium (10 g/L yeast extract, 20 g/L peptone, and 20 g/L dextrose).

### 3.2. Plasmid Construction for Yeast Surface Display of Linear Peptides

To express linear peptide sequences as Aga2p fusions, DNA encoding the linear peptide sequences was inserted into the pCTCON plasmid [51]. For example, the pCTCON-YESS-pep construct affording the expression of the model peptide ALQSGSRGGGEQK was constructed by amplifying gene block 1 with Pf1 and Pr1 and inserting the amplified product between the NheI and BamHI sites of pCTCON.

All double-stranded gene fragments were purchased from Integrated DNA Technologies (IDT). Primer oligonucleotides were acquired from IDT or Eton Biosciences. Primer sequences and gene block fragments are described in Tables S1 and S2, while randomized library oligonucleotides are detailed in Table S3. PCR amplification was performed using Phusion Polymerase (Thermo Fisher Scientific, Waltham, MA, USA) following the manufacturer’s protocols. Restriction digests of plasmid backbones and PCR products were executed at 37 °C for 2 h using a 5X excess of each restriction enzyme. Digested plasmid backbones were incubated with Antarctic phosphatase (New England Biolabs, Ipswich, MA, USA) for 1 h at 37 °C. Digested plasmids and PCR products were purified using a 9K series gel and PCR extraction kit (BioBasic, Markham ON, Canada). Overnight ligations using T4 DNA ligase (Promega, Madison, WI, USA) were performed at 16 °C with the digested plasmid backbones and PCR product inserts. Ligations were transformed into chemically competent Novablue *E. coli* cells. The cells were made chemically competent using Mix&Go! *E. coli* transformation buffers (Zymo Research, Irvine, CA, USA). The GeneJET™ plasmid miniprep kit (Thermo Fisher Scientific, Waltham, MA, USA) was used to harvest plasmids from overnight *E. coli* cultures.

### 3.3. Extracellular Treatment of Yeast Cells Displaying Linear Peptide Precursors with Transglutaminase

Lyophilized recombinant microbial transglutaminase (Zedira, Darmstadt, Germany) was reconstituted to a concentration of 12.8 mg/mL in PBS, pH 6. Aliquots of 10^8^ cells were washed with PBS, pH 6, resuspended in 500 μL of PBS, pH 6.0, and incubated overnight with transglutaminase (1 μM) at 37 °C under mild agitation. The cells were washed with 0.1% BSA PBS, pH 7.4 (0.1% PBSA) to remove any excess transglutaminase.

### 3.4. Evaluation of Peptide Cyclization Mediated by Extracellular Transglutaminase via Flow Cytometry

Yeast cells displaying linear peptide precursors (model sequence ALQSGSRGGGKS or 8-mer library sequences) fused to specific epitope tags (HA and c-myc tags for model sequence ALQSGSRGGGKS; HA and His tags for library cells) were induced. A portion of the induced cells was treated with soluble transglutaminase overnight. Untreated cells were incubated with PBS, pH 6 overnight at 37 °C. Flow cytometry analysis was performed on treated and untreated cells to measure the binding of antibodies targeting the epitope tags. For each sample, 2 × 10^6^ cells were labeled with a 1:100 dilution of either a chicken anti-c-myc antibody (Invitrogen, Carlsbad, CA, USA), a rabbit anti-HA antibody (Thermo Fisher Scientific, Waltham, MA, USA), or an anti-His-647 antibody (Qiagen, Germantown, MD, USA) for 15 min at 4 °C. The anti-His labeling was conducted in the dark. The non-fluorescently conjugated primary antibodies were detected by labeling with a 1:250 dilution of goat-anti-chicken antibody (Immunoreagents, Raleigh, NC, USA) or donkey-anti-rabbit (Immunoreagents, Raleigh, NC, USA) labeled with Alexa Fluor 488 (green), respectively. All secondary labeling took place in 50 μL of 0.1% PBSA, on ice and in the dark for 15 min. Washes with 0.1% PBSA were performed after each incubation. At least 50,000 cells from each sample were analyzed using a MACSQuant VYB Cytometer (Miltenyi Biotec, Bergisch Gladbach, Germany)).

Yeast cells displaying linear peptide precursors were also exposed to TEV protease after treatment with extracellular transglutaminase to evaluate if the modification afforded by transglutaminase affects access of the TEV protease to its cleavage site expressed within the peptide fusion constructs. Specifically, 10^8^ transglutaminase-treated yeast cells were washed 3X with PBS, pH 8 supplemented with 150 mM NaCl (PBSCl). The cells were then incubated overnight with TEV (3 μM) in PBSCl at 37 °C under mild agitation. The cells were then washed 3X with 0.1% PBSA and analyzed via flow cytometry as previously described. For comparison, (i) cells treated with transglutaminase but not TEV protease as well as (ii) cells treated with TEV protease but not transglutaminase were also analyzed via immunofluorescent labeling; also considered were cells left completely untreated. Cells that did not undergo a specific treatment were resuspended in the buffer used for the corresponding incubation period (PBS, pH 6 in lieu of the treatment with transglutaminase or PBSCl in lieu of the treatment with TEV protease).

### 3.5. Expression, Purification, and Biotinylation of Recombinant N-Terminal YAP, WW Domains of YAP, and TEV

The plasmid pET22b(+) was utilized for recombinant expression of YAP and its WW domains. Specifically, gene block 2 was amplified with primers Pf2 and Pr2 and inserted between the NdeI and XhoI sites of pET22b(+) to create plasmid pET22b(+)-WW, which affords the expression of the WW domains of YAP (AAs 165–271). In a similar manner, gene block 2 was amplified with primers Pf3 and Pr3 and inserted between the NdeI and XhoI sites of pET22b(+) to create plasmid pET22b(+)-YAP, which affords the expression of N-terminal YAP (AAs 1–291). For expression, positive clones were transformed into Rosetta *E.coli* cells and grown in 2XYT media (10 g/L tryptone, 10 g/L yeast extract, 5 g/L NaCl) plus 1x ampicillin. A 1 L culture of 2XYT was inoculated with a 5 mL overnight culture grown at 37 °C. After reaching an OD_600_ between 0.6 and 0.8, expression was induced using 0.5 mM isopropyl β-D-1-thiogalactopyranoside (IPTG) and allowed to proceed overnight at 20 °C. Expression of TEV protease in Rosetta *E. coli* cells using plasmid pRK793 (Plasmid No. 8827, Addgene, Watertown, MA, USA) was performed as previously described [52,53].

Each protein was purified using a Biologic LP FPLC system (Bio-Rad, Hercules, CA, USA) by metal affinity chromatography. Briefly, the cells were collected and sonicated for lysis in 35 mL of Buffer A-IMAC (20 mM HEPES, 137 mM NaCl, pH 7.8). The cell lysate was loaded onto a 5 mL Bio-Scale™ Mini-Profinity™ IMAC column (Bio-Rad, Hercules, CA, USA), washed with 50 mL of Buffer C-IMAC (20 mM HEPES, 800 mM NaCl, pH 7.8), flushed with 50 mL of Buffer A-IMAC, and eluted over a 50 mL linear gradient of Buffer B-IMAC (20 mM HEPES, 137 mM NaCl, 500 mM Imidazole, pH 7.8). The fractions were analyzed by sodium dodecyl sulfate (SDS)-polyacrylamide gel electrophoresis (PAGE) and fractions containing the protein of interest were pooled for dialysis into 20 mM HEPES, 150 mM NaCl, pH 7.8 (HEP) using SnakeSkin™ Dialysis Tubing (MWCO 3.5 kDa, Thermo Fisher Scientific, Waltham, MA, USA) prior to biotinylation. Proteins were biotinylated using the EZ-Link Sulfo-NHS-LC-Biotinylation kit (Thermo Fisher Scientific, Waltham, MA, USA) following the manufacturer’s protocol. Excess biotin was removed via dialysis into HEP buffer.

### 3.6. Construction of Yeast Display Library of Linear Peptide Precursors for Putative Cyclization by Extracellular Transglutaminase

The plasmid backbone (pCTCON-TG-8mer-template) was generated by amplifying Gene block 3 (Table S2) with primers Pf4 and Pr4 and inserting the amplified product between the AgeI and XhoI sites of pCTCON. An aliquot of 40 μg of pCTCON-TG-8mer-template was linearized using SalI and BamHI. DNA oligo 1 (Table S3) was amplified using primers Pf5 and Pr5. DNA oligo 1 encodes a linear peptide sequence containing eight randomized amino acid positions (NNK) flanked by residues A-L-Q and K, which are required for the transglutaminase-mediated modification. The digested backbone and the amplified product from oligo 1 were purified using phenol:chloroform extraction followed by DNA concentration using ethanol precipitation as described in [52]. The yeast library was created using a lithium aetate transformation protocol described in [54]; specifically, 12 μg of amplified product from oligo 1 and 4 μg of linearized pCTCON-TG-8mer-template vector were transformed into 400 μL of EBY100 via electroporation using a Bio-Rad Gene Pulser system; the electroporation conditions utilized were 2.5 KV, 25 μF, and 250 Ω. Four transformations in total were performed as well as a vector only control. The diversity of the resultant library was estimated to be~2.6·10^7^ as determined by plating serial dilutions of the combined transformation reactions onto SDCAA plates (20 g/L dextrose, 5 g/L casamino acids, 6.7 g/L yeast nitrogen base, 182 g/L sorbitol, 5.4 g/L Na_2_HPO_4_, 8.6 g/L NaH_2_PO_4_ ∙ H_2_O, and 12 g/L Agar).

### 3.7. Screening of a Yeast Display Library of Linear Peptide Precursors Putatively Cyclized via Extracellular Transglutaminase to Identify Cyclic Peptide Binders for N-Terminal YAP and Its WW Domains

The yeast display library of linear peptide precursors treated with extracellular transglutaminase was subjected to two rounds of magnetic selection and two rounds of FACS to identify binders to N-terminal YAP and its WW-domains. For each selection round, the library cells were induced and incubated with soluble transglutaminase. The incubation of the library cells with transglutaminase was scaled linearly for the number of library cells required for each selection round.

For magnetic selection, 25 μL of washed Biotin Binder Dynabeads (Thermo Fisher Scientific, Waltham, MA, USA) were precoated with 17 μg of biotinylated N-terminal YAP or WW-YAP overnight at 4 °C in 100 μL of 0.1% PBSA. In the first round of magnetic selection, a negative selection was performed by incubating 10^9^ library cells with 25 μL of non-functionalized Biotin Binder Dynabeads at room temperature for 1 h. Unbound cells were isolated using a magnet, and an additional negative selection was performed using the unbound yeast population and a fresh aliquot of non-functionalized beads. The unbound cells were then removed and utilized in a positive selection against the beads pre-coated with target protein for 1 h at room temperature. The magnetic beads were then separated from the unbound cells and washed 3X with 0.1% PBSA. Library cells positively bound to the target-functionalized magnetic beads were expanded in SDCAA media at 30 °C for 2 days. All magnetic selections took place in 4 mL of 0.1% PBSA. Prior to incubation with library cells, beads were washed 3X with 0.1% PBSA. The second round of magnetic selection was performed in a similar manner using only 10^8^ library cells. No negative selections were performed prior to the second magnetic selection.

The pool of cells obtained from the second magnetic selection were treated with transglutaminase and subjected to FACS using a FACSAria II cell sorter (Becton Dickinson, Franklin Lakes, NJ, USA). Specifically, 10^7^ library cells were labeled with biotinylated N-terminal YAP or WW-YAP for 1 h on ice. The cells were labeled with 1 μM of target protein during the first round of FACS, while 100 nM of target protein was utilized in the second round. The cells were then labeled with a 1:250 dilution of streptavidin R-phycoerythrin conjugate (SA-PE; Invitrogen, Carlsbad, CA, USA) for 15 min on ice in the dark; all labeling was performed in 100 μL of 0.1% PBSA. After each incubation, washes with 0.1% PBSA were performed. The cells isolated from the second round of FACS were plated onto SDCAA plates. The plasmids from individual clones were extracted from yeast culture using a Zymoprep™ Yeast Plasmid Miniprep II kit (Zymo Research, Irvine, CA, USA) and transformed into electrocompetent Novablue *E.coli* cells. Finally, the DNA was extracted from the overnight *E. coli* cultures and sequenced.

### 3.8. Estimation of Binding Affinity via Yeast Surface Titrations

The apparent binding affinity (*K_D_*) between yeast-displayed peptides (linear precursor or transglutaminase-treated) and a soluble protein (WW domains of YAP, N-terminal YAP, or bovine serum albumin) was estimated using a yeast surface titration method. Yeast cells encoding the linear precursor QLYLAYPAHK were induced. A portion of these cells was treated with transglutaminase as previously described. An aliquot of 0.2 × 10^7^ cells was incubated with varying concentrations of biotinylated WW-YAP, N-terminal YAP, or bovine serum albumin (BSA) for 1 h at 4 °C, followed by labeling with SA-PE (1:250 dilution) for 12 min on ice. All labeling took place in 50 μL of 0.1% PBSA, while washes with 0.1% PBSA were performed after each incubation. The cells were then analyzed by flow cytometry as described in [21,50]. A SA-PE only labeling was also carried out to quantify the mean background fluorescence. BSA was biotinylated. The values of *K_D_* were estimated using Equation (1):

(1) $$F=\frac{F_{max}{\left[L\right]}_{o}}{K_{D}+{\left[L\right]}_{o}}$$

where *F* is the background subtracted mean fluorescence intensity, [*L*]*_o_* is the concentration of soluble target protein used for labeling, and *F_max_* is the background subtracted fluorescence intensity when surface-saturation is obtained. *F_max_* was set as the highest value of fluorescence observed in each data set. Data from each independent replicate were normalized to their corresponding *F_max_* value. For each repeat, the normalized fluorescence values were fit to Equation (1) to estimate *K_D_* by minimizing the error. The average *K_D_* of three independent repeats is reported plus or minus a standard deviation. The Hook effect was observed at high antigen concentrations (i.e., the fluorescence signal decreased at high levels of [*L*]*_o_*) [55]. Accordingly, adsorption data obtained for high levels of [*L*]*_o_*, where the fluorescence signal decreased, were excluded from fitting.

### 3.9. Synthesis of Peptides Cyclo[E-LYLAYPAH-K] and RYSPPPPYSSHS (PTCH) on Toyopearl Resin

Peptides were synthesized on Toyopearl AF-Amino-650 M resin (functional density ~0.2 meq/mL, Tosoh, Tokyo, Japan) using a Alstra Initator+ automated synthesizer (Biotage, Uppsala, Sweden) via Fmoc/tBu chemistry [56,57]. All amino acids couplings were conducted with 5 equivalents of amino acid (0.5 M in anhydrous dimethylformamide, DMF), 4.95 eq. 2-(6-Chloro-1H-benzotriazole-1-yl)-1,1,3,3-tetramethylaminium hexafluorophosphate (HCTU, 0.5 M in DMF) and 10 eq. diisopropylethylamine (DIPEA, 2 M in NMP) for 5 min at 75 °C [58]. Fmoc deprotection was achieved by incubating the resin twice in 20% piperidine in DMF (*v*/*v*) at room temperature for 10 min. All Fmoc-protected amino acids, HCTU, piperidine, and anhydrous solvents were from Chem-Impex International, Inc (Wood Dale, IL, USA). The cyclization of ELYLAYPAHK was performed as described in [59]. Briefly, the allyl ester (OAll) protection was removed from N-terminal Glu(OAll) using Tetrakis(triphenylphosphine)palladium(0) in DCM, while the methyltrityl (Mtt) protection was removed from C-terminal Lys(Mtt) in 2% trifluoroacetic acid (TFA) and 5% triisopropylsilane (TIPS) in DCM [60]. The conjugation of the free carboxyl group of Glu and the amino group of Lys via an amide bond was performed by incubating the resin twice with 1 eq. HCTU (0.5 M in DMF) and 2 eq. DIPEA (2M in NMP) for 20 min at 75 °C. All reagents utilized for site-selective deprotection and peptide cyclization were acquired from Millipore Sigma (Burlington, MA, USA). Both peptides were rinsed copiously with DCM and deprotected by acidolysis using a 90/5/3/2 TFA/thioanisole/ethanedithiol/anisole cocktail at 10 mL per gram of resin for 2 h at room temperature. The resin was then washed copiously with DCM and DMF and dried under nitrogen.

### 3.10. Evaluation of Binding Capacity, Affinity, and Selectivity of Peptide Functionalized Resin for N-Terminal YAP and WW-YAP

Aliquots of 1 mg of cyclo[*E-*LYLAYPAH*-K*]-Toyopearl resin and RYSPPPPYSSHS-Toyopearl resin were washed 3X with PBS, pH 7.4 and incubated for 1 h at room temperature with 400 μL of the target protein—either N-terminal YAP or WW-YAP—at various concentrations (20 μg/mL to 3 mg/mL) in PBS. The supernatants were collected and analyzed by UV spectrophotometry at 280 nm to measure the amount of unbound protein. The amount of protein adsorbed on the resin was determined via mass balance. The *K_D_* describing the binding affinity of the soluble protein to the peptide-functionalized resin was determined by fitting the adsorption data to a Langmuir isotherm:

(2) $$q=\frac{q_{max}\left[C\right]}{K_{D}+\left[C\right]}$$

where *q* is the amount of protein adsorbed on the resin (mg protein/mL resin), [*C*] is the unbound concentration of protein at equilibrium (mg protein/mL solution), *q_max_* is the maximum protein binding capacity (mg protein/mL resin), and *K_D_* is the dissociation constant (mg protein/mL solution). Equation (2) was independently fit to the data obtained from triplicate adsorption studies, and the fitted values of *K_D_* and *q_max_* were averaged across the repeats. The binding of BSA and lysozyme (13 μg/mL to 2 mg/mL in HEP buffer) to the cyclo[*E-*LYLAYPAH*-K*]-Toyopearl resin was evaluated in a similar manner and compared to the binding of N-Terminal YAP and WW-YAP for similar values of equilibrium protein concentration [*C*].

### 3.11. In Silico Modeling of WW:Peptide Interactions

The crystal structures of the WW1 and WW2 domains of human YAP (PDB: 2LTV and 2LTW, respectively) [47] were prepared using Schrödinger’s ProteinPrep Wizard (Release 2020-1, Schrödinger Inc., New York, NY, USA) [61,62] to correct missing atoms and/or side chains, add explicit hydrogens, assign tautomeric states, optimize hydrogen bonding networks, and minimize the energy of both target proteins using the OPLS3e force field [63]. Peptides cyclo[*E-*LYLAYPAH*-K*] and QLYLAYPAHK were designed in Avogadro (Version 1.2, Avogadro Chemistry, Pittsburgh, PA, USA) [64,65] and equilibrated via molecular dynamics in GROMACS (Version 2020.1, GROMACS Development Team) [66,67,68] using the OPLS all-atom force field [69,70,71]. Briefly, each peptide was placed in a simulation box with periodic boundary conditions containing 1000 water molecules (TIP3P water model) [72], minimized with 10,000 steps of steepest gradient descent, heated to 300 K in an NVT ensemble for 500 ps with 2 fs time steps, and equilibrated to 1 atm with a NPT simulation for 500 ps with 2 fs time steps. The production run for every peptide was performed in the NPT ensemble at constant T = 300 K using the Nosé-Hoover thermostat [73,74,75] and *p* = 1 atm using the Parrinello-Rahman barostat [76,77]. The leap-frog algorithm was used to integrate the equations of motion with integration steps of 2 fs. All covalent bonds were constrained using the LINCS algorithm [78]. The short-range electrostatic and Lennard-Jones interactions were calculated using a cutoff of 1.2 nm, and the long-range electrostatic interactions were treated using the particle-mesh Ewald method [79,80,81]. The peptides were then docked in silico on the WW1 and WW2 domains of human YAP using the docking software HADDOCK (High Ambiguity Driven Protein-Protein Docking, Version 2.2, Alexandre Bonvin, Utrecht University, Utrecht, Netherlands) [48,82,83]. All residues on WW1 and WW2 domains with solvent accessibility >50% were defined as “active”, and—given the small size of the targets—all other residues were defined as “passive”. All amino acids within the peptide ligands were denoted as ”active”, except for a GSG tripeptide spacer that was defined as not involved in the interaction to simulate the directionality of binding. The HADDOCK process was articulated in three steps: (i) rigid docking for 1000 structures, (ii) semi-flexible docking for 200 structures, and (iii) water-refined fully flexible docking for 200 structures. The resulting docked structures were clustered using ProFit (Version 3.1, Martin, A.C.R, London, UK). All the poses in the clusters were evaluated using the scoring function MM-PBSA [84] to select binding poses to be refined using atomistic molecular dynamics (MD) simulations. Briefly, the WW1:peptide and WW2:peptide complexes were solvated with 30,000 TIP3P water molecules in a cubic periodic box of 10 nm side lengths. The MD simulations were conducted at 300 K and 1 atm using the Amber99SB force field. The MM/GBSA algorithm was used to evaluate the resultant WW1:peptide and WW2:peptide complexes [85,86].

## 4. Conclusions

In this work, we have demonstrated the use of enzymatic modification by transglutaminase as an efficient route to produce yeast display libraries of structurally modified peptides. Enzymatic cyclization is particularly attractive, especially as compared to traditional strategies relying on disulfide linkage or promiscuous chemical crosslinkers, since enzymes enable site-selective ligation through recognition of specific peptide motifs. In this study we explored the use of transglutaminase to cyclize peptides displayed on the surface of yeast cells, given the versatility of this enzyme in modifying peptides with varying length and composition provided that the appropriate flanking residues are present. To this end, we have developed two routes to achieve the display of cyclic peptide sequences on the surface of yeast, one employing extracellular transglutaminase and one relying on the intracellular transglutaminase-mediated modification of linear peptide constructs in the endoplasmic reticulum. For each route, we have designed peptide yeast display constructs to optimize the yield of peptide modification and developed indirect assays that rely on the detection of epitope tags to assess the degree of putative cyclization of single model or multiple combinatorial peptides. While the alleged peptide cyclization via intracellular catalytic amidation afforded a relatively low efficiency, its extracellular counterpart performed with soluble transglutaminase proved significantly more efficient. Nonetheless, important findings were made to optimize the concurrent intracellular expression and trafficking of transglutaminase and the linear peptide precursors to improve the yield of putative cyclization. In this regard, future work will focus on further optimizing the integration of transglutaminase-mediated modification with the yeast display platform to accomplish the efficient display of cyclic peptides for de novo discovery of bioactive peptides. In this context, this study has shown the successful application of magnetic and fluorescent based selections to the yeast display platform for the identification of cyclic peptides that selectively target WW-YAP and N-terminal YAP. The discovery and characterization methods presented in this work are agnostic to the cyclization chemistry utilized and can be used to characterize other enzyme-based cyclization strategies. Accordingly, we expect that this platform will prove a flexible tool for high throughput engineering of peptide-based ligands and drugs.

## Figures and Tables

**Table 1:** Values of binding affinity (K_D_) of protein:peptide complexes estimated from surface titrations of peptide-displaying yeast cells with soluble protein; N/A: not applicable; N/D: not detectable; L/B: low binding. The average K_D_ of three independent replicates plus/minus one standard error is reported.

Peptide Ligand	K_D_ (μM)		
	WW-YAP	N-YAP	BSA
cyclo[*E*-LYLAYPAH-*K*]	0.68 ± 0.21	1.75 ± 0.33	N/D
QLYLAYPAHK	L/B	0.74 ± 0.15	N/A

**Table 2:** Values of binding affinity (K_D_) and capacity (Q_max_) of cyclo[*E*-LYLAYPAH-*K*] and PTCH peptide conjugated on Toyopearl resin for the WW domains of YAP (WW-YAP) and N-terminal YAP (N-Yap). The isotherm binding data presented in Figure 5 were fit to a Langmuir isotherm to estimate K_D_ and Q_max_ values. The average K_D_ and Q_max_ of three independent replicates plus/minus one standard error is reported.

Peptide Ligand	Target Protein			
	WW-Yap		N-YAP	
	K_D_ (μM)	Q_max_ (mg/mL)	K_D_ (μM)	Q_max_ (mg/mL)
cyclo[*E*-LYLAYPAH-*K*]	2.64 ± 0.71	5.60 ± 0.42	0.34 ± 0.02	3.03 ± 0.13
PTCH peptide	2.88 ± 0.48	8.3 ± 0.27	0.20 ± 0.07	4.11 ± 0.12

## Data Availability

Data is contained within the article or Supplementary Materials.

## Supplementary Materials

The following are available online at https://www.mdpi.com/1422-0067/22/4/1634/s1. Figure S1: Co-expression of a linear peptide construct and transglutaminase to achieve the putative cyclization of the model peptide either intracellularly, during trafficking through the endoplasmic reticulum, or upon display on the surface of the yeast cell, Figure S2: Engineering of the pCTCON plasmid to express concurrently the model peptide sequence (ALQSGSRGGG) fused to the c-myc epitope sequence (EQKLISEEDL) and the full-length transglutaminase enzyme, Figure S3: Engineering of pCTCON plasmid, Figure S4: Mean fluorescence intensity (MFI) of engineered constructs, Figure S5: Representative flow cytometry plots comparing the binding of fluorescently labeled antibodies to the HA (x-axis) and c-myc (y-axis) tags displayed, Figure S6: Screening of a yeast display library of peptides putatively cyclized by active transglutaminase expressed intracellularly, Figure S7: Yeast surface titrations of N-terminal N-YAP (N-YAP, black circles) and BSA (green diamonds) on cells, Table S1: Oligonucleotide primers, Table S2: Gene Blocks, Table S3: Oligonucleotides encoding randomized peptides.
