# Supplementary material for: Screening of Yeast Display Libraries of Enzymatically Treated Peptides to Discover Macrocyclic Peptide Ligands
Source: Int J Mol Sci. 2021 Feb 5;22(4):1634. doi: 10.3390/ijms22041634 (PMC7915732; doi:10.3390/ijms22041634)
Supplement: Supplementary file 1 [file ijms-22-01634-s001.pdf]

Screening of Yeast Display Libraries of Enzymatically Treated Peptides to Discover Macrocyclic Peptide Ligands

John Bowen, John Schneible, Kaitlyn Bacon, Collin Labar, Stefano Menegatti and Balaji M. Rao

S.1. Supplemental Methods

S.1.1. Plasmid construction for intracellular expression of transglutaminase and display of linear peptide precursors on the surface of yeast cells

The pCTCON-YESS-pep construct was modified to enable expression of the transglutaminase enzyme downstream of the Gal10 promoter. To this end, gene block 4 was amplified with Pf6 and Pr6, and inserted between the AgeI and KpnI sites of pTCTON-YESS-pep (*Section 2.2*) to create pCTCON-YESS-Full-Length-TG-dual-expression ("Full Length") construct. The latter affords co-expression of the linear peptide ALQSGSRGGGEQK under the direction of the Gal1 promoter and transglutaminase under the direction of the Gal10 promoter.

S.1.2. Modification of the plasmids for intracellular expression of transglutaminase

The pCTCON-YESS-Pro-KR-Active-TG-dual-expression vector ("Pro-Kex-TG") was constructed via amplification of the "Full Length" construct (*Section S.1.1*) using primers Pf7 and Pr7, which inserted a 6 base pair oligonucleotide encoding the Kex2 recognition site between the pro and active transglutaminase sequences. An aliquot of 50 ng of purified PCR product was treated with 1 μL of T4 polynucleotide kinase and 1 μL of T4 DNA ligase (New England Biolabs) for 1 h at room temperature followed by incubation for 20 min at 65 °C. The DNA was then treated for 5 min at room temperature with 1 μL of DpnI (New England Biolabs) prior to transformation into electrocompetent Novablue *E. coli* cells (EMD Millipore). The pCTCON-YESS-Active-TG-dual-display construct ("active TG, lead/c-myc") was produced by amplifying the pCTCON-YESS-Full-Length-TG-dual-expression vector using primers Pf8 and Pr8, which removed the pro-transglutaminase sequence prior to the treatments with kinase, ligase, and DpnI.

A similar process was used to introduce a point mutation in the transglutaminase substrate sequence, wherein the glutamine (Q) residue was replaced with an alanine (A) residue. Briefly, the pCTCON-YESS-Pro-KR-Active-TG-dual-expression vector was amplified using primers Pf9 and Pr9, followed by the treatments with kinase, ligase, and DpnI to yield vector pCTCON-YESS-Pro-KR-Active-TG-Q-to-A-SDM-dual-expression ("Pro-Kex-TG w/ Q-to-A SDM"). The "active TG, lead/c-myc" plasmid was also modified with a Q-to-A mutation by amplifying pCTCON-YESS-Active-TG-dual-display construct with primers Pf9 and Pr9 prior to kinase, ligase, and DpnI treatment to yield vector pCTCON-YESS-Active-TG-Q-to-A-SDM-dual-expression ("active TG, Q-to-A lead/c-myc").

S.1.3. Evaluation of intracellular transglutaminase-mediated peptide cyclization via flow cytometry

Yeast cells co-expressing linear peptide precursors and an intracellular form of transglutaminase (*i.e.*, "Full Length", "Pro-Kex-TG", "Pro-Kex-TG w/ Q-to-A SDM", "active TG, lead/c-myc", or "active TG, Q-to-A lead/c-myc") were induced for different time intervals (24, 48, 72, or 96 h). After

induction, a subset of the cells was treated with TEV protease as described in *Section 2.3* to evaluate the extent of putative peptide cyclization. Immunofluorescent labeling using anti-HA and anti-c-myc antibodies (Invitrogen) followed by flow cytometry analysis was performed as described in *Section 2.3* to detect the binding of antibodies to specific epitope tags. Induced cells that were not exposed to TEV protease were also analyzed for comparison.

S.1.4. Construction of a yeast display library of linear peptide precursors to be putatively cyclized by intracellular transglutaminase

The yeast display library co-expressing the linear peptide precursors and intracellular transglutaminase was produced as described in *Section 2.5*. Specifically, the pCTCON-YESS-Active-TG-dual-display construct was digested with NheI and XhoI restriction enzymes at 37 °C for 2 h. The DNA oligo 2 (Table S3) was amplified with primers Pf10 and Pr10; this DNA block encodes a linear peptide sequence containing seven randomized amino acid positions (NNK) framed between residues A-L-Q and K. The diversity of the resulting library was estimated to be ~ 1.6×10^7.

S.1.5. Screening of a yeast display library of linear peptide precursors putatively cyclized by intracellular transglutaminase to identify cyclic peptide binders for N-terminal YAP

Prior to every screening round, the pool of library mutants expressing intracellular transglutaminase was induced for 48 h at 20 °C. The library was screened against N-terminal YAP by performing one round of magnetic selection followed by one round of FACS (1 μM N-terminal YAP labeling), implementing the method described in *Section 2.6*. Cells isolated during the FACS screening round were plated onto SDCAA plates, and individual clones were selected for sequencing. The enrichment of peptide binders targeting N-terminal YAP between the screening rounds was evaluated via flow cytometry. An induced population of cells obtained from each screening round was labeled with biotinylated N-terminal YAP (500 nM) followed by SA-PE detection as described in *Section 2.6*.

S.1.6. Binding affinity estimation of peptides selected from intracellularly-expressed transglutaminase library via yeast surface titration

The yeast plasmid encoding cyclo[*E*-VQCRGKGEQ-*K*] (isolated from the intracellular transglutaminase library, as described in *Section 3.6*) was modified to mutate the substrate recognition site of transglutaminase and ensure the expression of the peptide in its linear form. Specifically, the point mutation strategy described in *Section S.1.2* was implemented to convert the glutamine (Q) residue to an alanine (A) residue, affording a plasmid encoding the surface display of the linear peptide sequence AVQCRGKGEQK.

Yeast surface titrations of N-terminal YAP were performed as described in *Section 2.7* on yeast cells expressing either cyclo[*E*-VQCRGKGEQ-*K*] or the linear peptide AVQCRGKGEQK to estimate the binding affinity (K_D) of the yeast-displayed peptides for N-terminal YAP. Control yeast surface titrations using biotinylated BSA were also carried out using yeast cells displaying cyclo[*E*-VQCRGKGEQ-*K*].

S.2. Supplemental Results and Discussion

S.2.1. Optimizing the expression of linear peptides putatively cyclized by intracellular transglutaminase

To circumvent the need for soluble transglutaminase, we evaluated an intracellular route to transglutaminase-mediated peptide cyclization (Figure S1). In the yeast display platform, the expression of peptides on the cell surface is controlled by the inducible galactose promoter Gal 1/10. The bidirectional nature of Gal 1/10 has been leveraged to achieve the concurrent expression of peptides and enzymes that can edit the chemical composition and structure of the expressed peptides. For example, the tobacco etch virus protease was engineered via the Yeast Endoplasmic Sequestration System (YESS) to achieve dual display of a TEV enzyme library in conjunction with specific and non-specific selection substrates [1]. In this approach, both enzyme and substrate transit through the endoplasmic reticulum (ER), where the enzyme can act on the substrate peptide construct prior to its display on the cell surface. In another example, the modifying enzyme Proc M and its lanthipeptide substrate were co-expressed resulting in the display of a macrocyclic lanthipeptide on the yeast surface [2]. In a similar fashion, we expressed transglutaminase under the control of the Gal10 promoter together with a model peptide substrate under the control of the Gal1 promoter (Figure S2A). The endoplasmic reticulum retention sequence FEHDEL [1] was added to the C-terminus of both the peptide and transglutaminase to increase their residence time within the ER by docking onto the ER membrane. We anticipated that this hybrid mechanism of intracellular trafficking and alleged peptide cyclization would afford the display of cyclic peptides on the yeast surface.

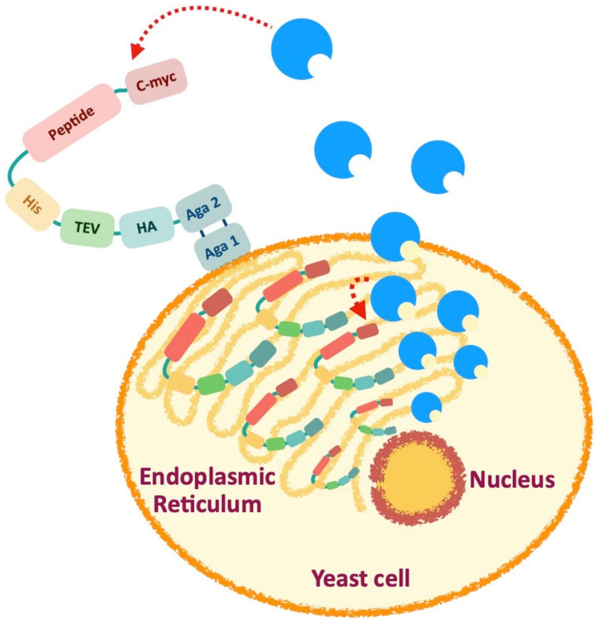

Figure S1. Co-expression of a linear peptide construct and transglutaminase to achieve the putative cyclization of the model peptide either intracellularly, during trafficking through the endoplasmic reticulum, or upon display on the surface of the yeast cell.

The model lead sequence ALQSGSRGGG fused to the c-myc epitope tag EQKLISEEDL, which contains a lysine (K) that can be modified by transglutaminase for cyclization, was used to evaluate this hypothesis. The full sequence of *Streptomyces mobaraensis* transglutaminase (GenBank accession number AAT65817.1, 395 AAs) was expressed under the Gal10 promoter; the protein comprises a 60 AA-long N terminal pro sequence and a 335-AA long transglutaminase enzyme. The

yield of the transglutaminase-mediated reaction was evaluated via immunofluorescent flow cytometry of the yeast cells – either following treatment with TEV protease or left untreated – via c-myc tag detection as described in *Section 3.1*. Upon induction at different time intervals (24, 48, 72, and 96 h), the cells were treated with TEV protease to attempt the cleavage of the peptide fusion from the yeast surface. The treatment with TEV protease consistently resulted in a significant reduction in the binding of the anti-c-myc antibody (Figures S2B–E), indicating that most of the displayed peptides are linear.

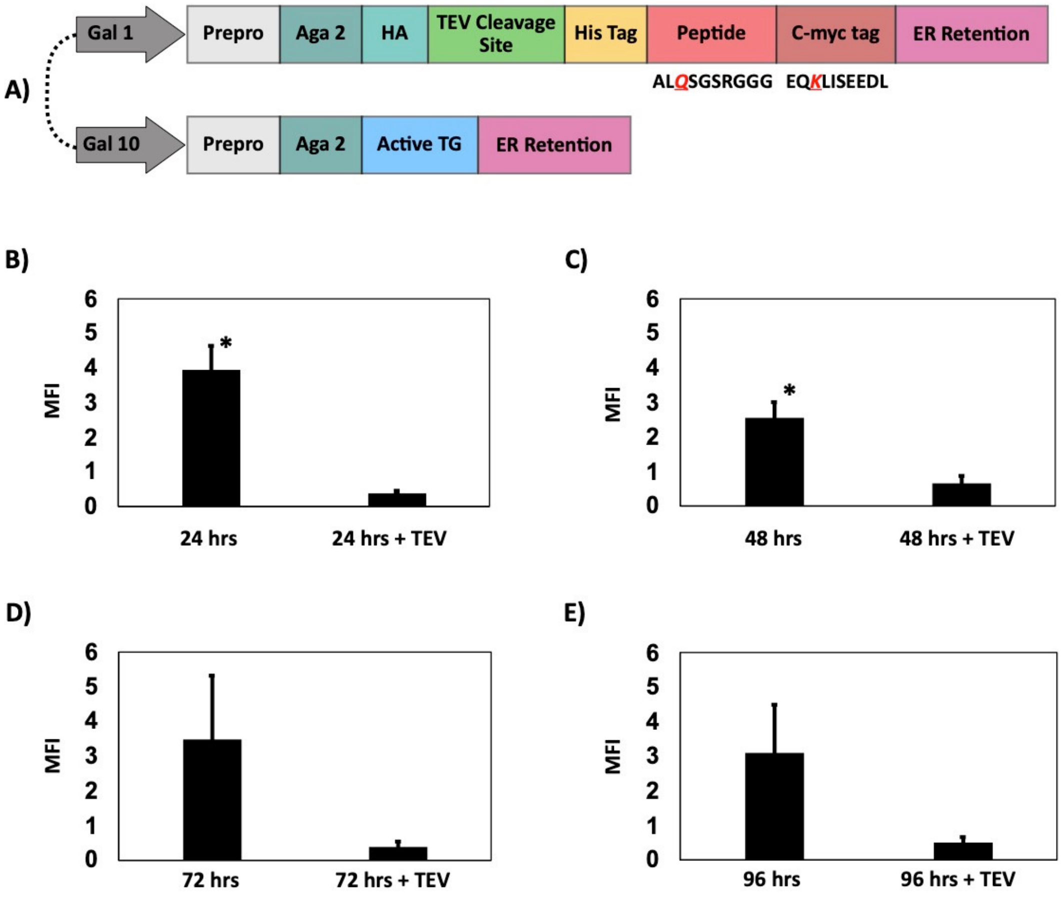

Figure S2. (A) Engineering of the pCTCON plasmid to express concurrently the model peptide sequence (ALQSGSRGGG) fused to the c-myc epitope sequence (EQKLISEEDL) and the full-length transglutaminase enzyme. Mean fluorescence intensity (MFI) of c-myc tag levels measured on yeast cells – either treated with TEV protease or left untreated – wherein the peptide construct and the full-length transglutaminase enzyme were co-expressed for **(A)** 24 hrs, **(B)** 48 hrs, **(C)** 72 hrs, and **(D)** 96 hrs. The error bars correspond to the standard error of the mean from three independent replicates. A two-tailed paired t-test was performed; * indicates $p < 0.1$ and ** indicates $p < 0.05$ in comparison to the cells treated with TEV protease.

To improve the yield of putative intracellular cyclization, we modified the structure of the encoded transglutaminase. The full-length enzyme contains a pro-peptide sequence that forms an α-helix into the catalytic site of transglutaminase and is typically cleaved by intracellular endopeptidases after translation and protein folding [3]. We hypothesized that this α-helix remains tethered to the folded enzyme, thus hindering its active site and preventing the cyclization of the model peptide.

To this end, two different transglutaminase variants were initially explored. First, we introduced a Kex2 endopeptidase cleavage recognition site (K-R) between the pro-peptide and the

transglutaminase sequence, which has been observed to promote the secretion of active transglutaminase in *Pichia pastoris* and *Candida boidinii* [3]. Kex2 peptidase is expressed endogenously in *Saccharomyces cerevisiae* and is present in the Golgi, which is part of the secretory pathway [4]. Next, we introduced a single point mutation into the substrate sequence by mutating the glutamine (Q) into an alanine (A). This mutation removes one of the two residues necessary for cyclization and enables a direct comparison between the different "intracellular" constructs. The construction of the "Pro-Kex-TG" vector, which contains the Kex2 protease recognition sequence, and the "Pro-Kex-TG w/ Q-to-A SDM" vector, which contains both the Kex2 protease recognition sequence and a "Q-to-A" mutation (SDM: site directed mutagenesis), is shown in Figures S3A and S3B.

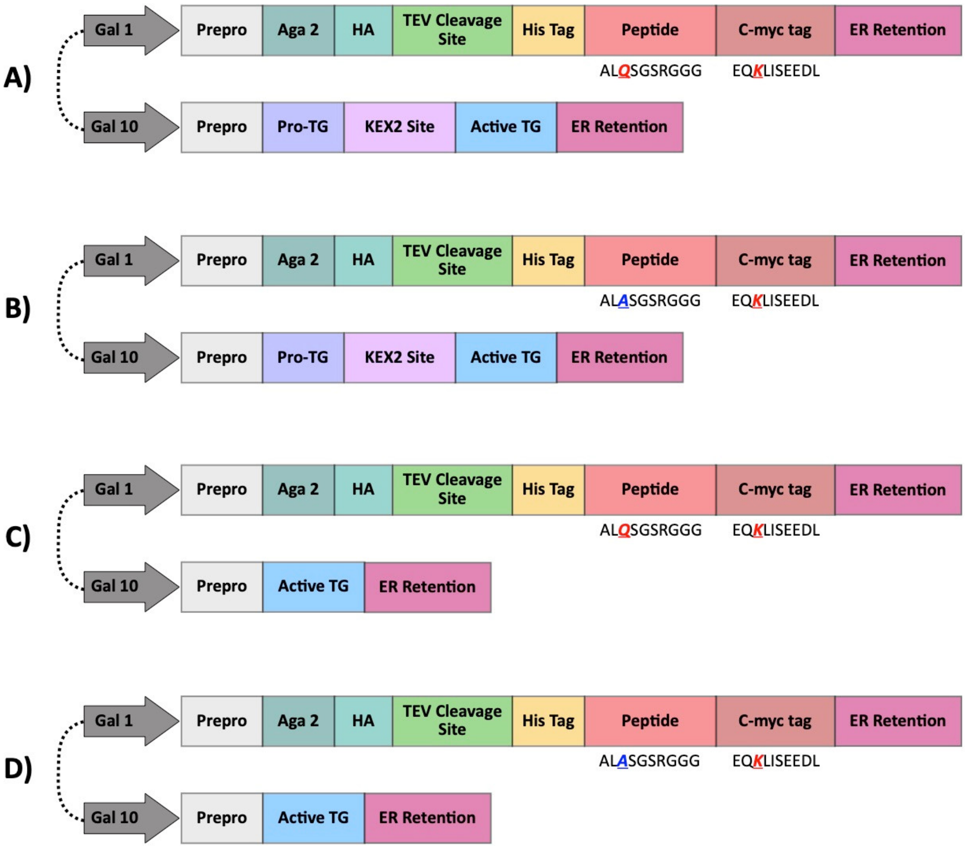

Figure S3. Engineering of pCTCON plasmid to express **(A)** the model peptide sequence ALQSGSRGGG fused to the c-myc epitope sequence EQKLISEEDL concurrently with transglutaminase containing a Kex2 endopeptidase recognition site (K-R) between its pro and active sequences ("Pro-Kex-TG"); **(B)** the mutated model peptide sequence ALASGSRGGG fused to the c-myc epitope sequence concurrently with transglutaminase containing a Kex2 endopeptidase recognition site between its pro and active sequences ("Pro-Kex-TG w/ Q-to-A SDM"); **(C)** the model peptide sequence fused to the c-myc epitope sequence concurrently with active transglutaminase ("active TG, lead/c-myc"); and **(D)** the mutated peptide sequence fused to the c-myc epitope sequence concurrently with active transglutaminase ("active TG, Q-to-A lead/c-myc").

To evaluate the efficiency of transglutaminase-mediated peptide modification, the induced peptide constructs were characterized via flow cytometry by measuring the binding of anti-HA and anti-c-myc antibodies to yeast cells that were either exposed to TEV protease or left untreated. During the analysis of the constructs treated with extracellular transglutaminase (*Section 3.2*) we observed that putative peptide cyclization reduces TEV proteolysis (Figure 2C); cells displaying transglutaminase-modified peptides, in fact, exhibited comparable low levels of c-myc expression irrespectively of TEV treatment. However, the exposure of cells harboring the "Pro-Kex-TG" construct to TEV protease caused a significant decrease in the detection of the c-myc tag (Figure S4A),

suggesting that the yield of cyclization using intracellular transglutaminase is rather modest. For comparison, we analyzed the binding of c-myc antibodies to yeast cells containing the "Pro-Kex-TG w/ Q-to-A SDM" plasmid, either exposed to TEV protease or untreated. The c-myc signal of the untreated "Pro-Kex-TG" construct was high and comparable to that of the untreated "Pro-Kex-TG w/ Q-to-A SDM" construct, indicating that low or no peptide cyclization was achieved when using the "Pro-Kex-TG" construct (Figure S4B). Had the "Pro-Kex-TG" construct indeed afforded the display of cyclic peptides, a difference in anti-c-myc antibody binding to the "Pro-Kex-TG" *vs.* "Pro-Kex-TG w/ Q-to-A SDM" cells would have been noted, since transglutaminase cannot modify the peptides expressed by the "Pro-Kex-TG w/ Q-to-A SDM" cells; in this context, we also observed a comparable expression of the HA tag on both constructs, confirming the display of the peptides on the surface of the yeasts harboring both constructs (Figure S4B).

We also examined whether the induction time influences the display of cyclic peptides when utilizing the "Pro-Kex-TG" construct. No detectable difference in HA or c-myc tag expression was observed between the cells harboring the "Pro-Kex-TG" or "Pro-Kex-TG w/ Q-to-A SDM" construct, whether induced for 24 hrs or 48 hrs and not exposed to TEV protease (Figure S5A). However, after TEV treatment, a marked decrease in c-myc signal was observed of the cells harboring both constructs and induced for either 24 or 48 hrs (Figure S5B), suggesting that an increase in induction time does not improve the cyclization efficiency. Collectively, these data indicate that the insertion of the Kex2 recognition sequence between the pro-peptide and active transglutaminase does not improve the cyclization efficiency.

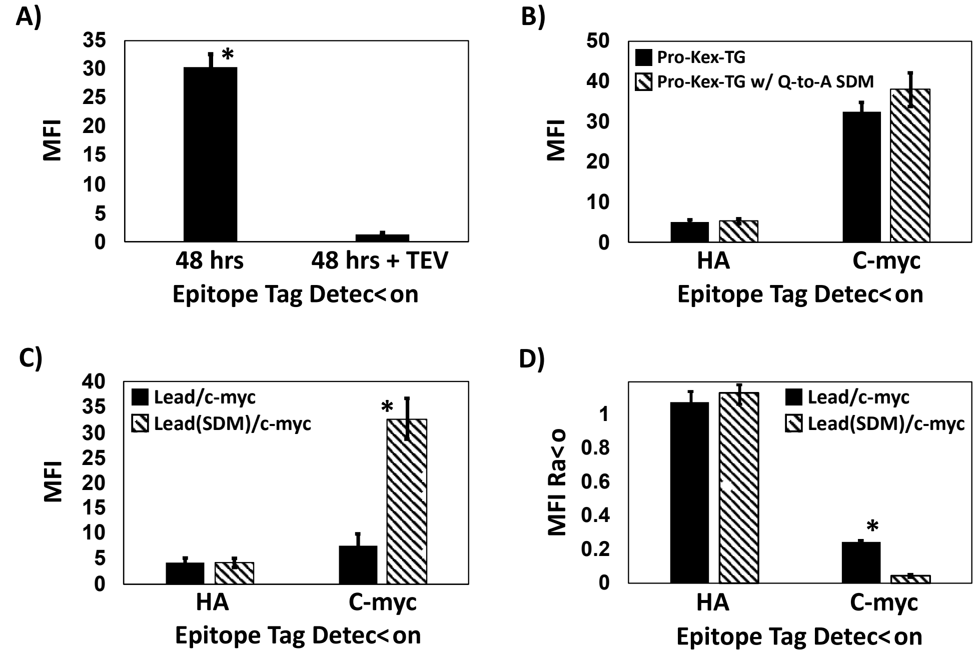

Figure S4. Mean fluorescence intensity (MFI) of **(A)** c-myc tag levels detected on yeast cells engineered with the "Pro-Kex-TG" vector, induced for 48 hrs, and either treated with TEV protease or left untreated; **(B)** HA and c-myc tag levels detected on yeast cells engineered with either the "Pro-Kex-TG" vector or the "Pro-Kex-TG w/ Q-to-A SDM" vector and induced for 48 hrs (no treatment with TEV protease); and **(C)** HA and c-myc tag levels detected on yeast cells induced for 48 hrs to express either the "active TG, lead/c-myc" or the mutated "active TG, Q-to-A lead/c-myc" construct (no treatment with TEV protease) **(D)** MFI ratio comparing the HA and c-myc tag levels detected on yeast cells treated with TEV protease to those left untreated. The cells were induced for 48 hrs to express either the "active TG, lead/c-myc" or the mutated "active TG, Q-to-A lead/c-myc" construct. The MFI Ratio was calculated by dividing the MFI measured on cells treated with

TEV protease with the MFI measured on untreated cells. The error bars correspond to the standard error of the mean from three independent replicates. A two-tailed paired t-test was performed, where * indicates $p < 0.05$.

We therefore sought an alternative route where we expressed active transglutaminase (AAs 61–395) without the pro-peptide sequence under the control of the Gal10 promoter concurrently with the c-myc epitope tag EQKLISEEDL downstream of either the lead sequence ALQSGSRGGG ("active TG, lead/c-myc") or the mutated sequence ALASGSRGGG (active TG, Q-to-A lead/c-myc) under the control of the Gal1 promoter (Figures S3C,D). Both cell populations were induced for 48 hrs, treated with or without TEV protease, and analyzed via immunofluorescent flow cytometry to detect the binding of antibodies targeting the HA and c-myc tags (Figures S4C,D; representative histograms are shown in Figures S5C,D). As anticipated, the HA signal detected on cells harboring the "active TG, lead/c-myc" construct did not differ significantly upon TEV protease treatment (Figure S4D).

A comparison between the binding of anti-c-myc antibodies to cells harboring the "active TG, lead/c-myc" *vs.* "active TG, Q-to-A lead/c-myc" constructs (Figure S4C) suggests that putative cyclization of the peptides expressed by the "active TG, lead/c-myc" construct has occurred. As detailed in *Section 3.2*, the ligation of the K residue of the c-myc tag to the Q residue of the lead sequence by transglutaminase likely results in a reduction of anti-c-myc antibody binding to the displayed modified peptide. Moreover, the fluorescent c-myc signal obtained with the "active TG, lead/c-myc" construct was found to be 4-fold lower than that of the other two constructs ("full-length" and "Pro-Kex-TG") and comparable to the signal obtained when analyzing cells displaying the peptide cyclized by treatment with extracellular transglutaminase (Figure 2A). These results collectively indicate that expression of active transglutaminase without the pro-peptide affords the cyclization of a portion of the expressed linear peptides. This was further corroborated after measuring the binding of anti-c-myc antibodies to cells harboring the "active TG, lead/c-myc" construct that were exposed to TEV protease. Following TEV treatment, the cells harboring the "active TG, Q-to-A lead/c-myc" plasmid showed significantly lower binding of anti-c-myc antibodies than cells harboring the "active TG, lead/c-myc" plasmid (Figure S4D); the Q-to-A peptide mutation encoded by the "active TG, Q-to-A lead/c-myc" plasmid, in fact, prevents alleged peptide cyclization, allowing TEV proteolysis to proceed unhindered. Observing a larger loss of anti-c-myc antibody binding to cells harboring the linear construct ("active TG, Q-to-A lead/c-myc") *vs.* those harboring the active transglutaminase ("active TG, lead/c-myc") construct, upon exposure to TEV protease, further corroborates the claim that the latter affords peptide cyclization.

Notably, conclusive differences in fluorescence signal ("active TG, lead/c-myc" *vs.* "active TG, Q-to-A lead/c-myc") were observed only with cells induced for 48 h, whereas little-to-no variation was observed with cells induced for 24 h (Figures S5C). This suggests the need for a sufficiently high level of transglutaminase in the ER to achieve detectable putative peptide cyclization. To this end, we included an ER-docking sequence downstream of the transglutaminase and peptide sequences in the plasmid construct to increase the residence time of both molecules and the probability of interaction. Transglutaminase is ultimately secreted from the yeast into the supernatant, where it may catalyze additional (extracellular) cyclization of the peptides displayed on the surface of the cells. Collectively, these results show that the co-expression of a substrate peptide sequence and active transglutaminase lacking its pro-peptide sequence affords the display of putatively cyclized peptides on the yeast surface.

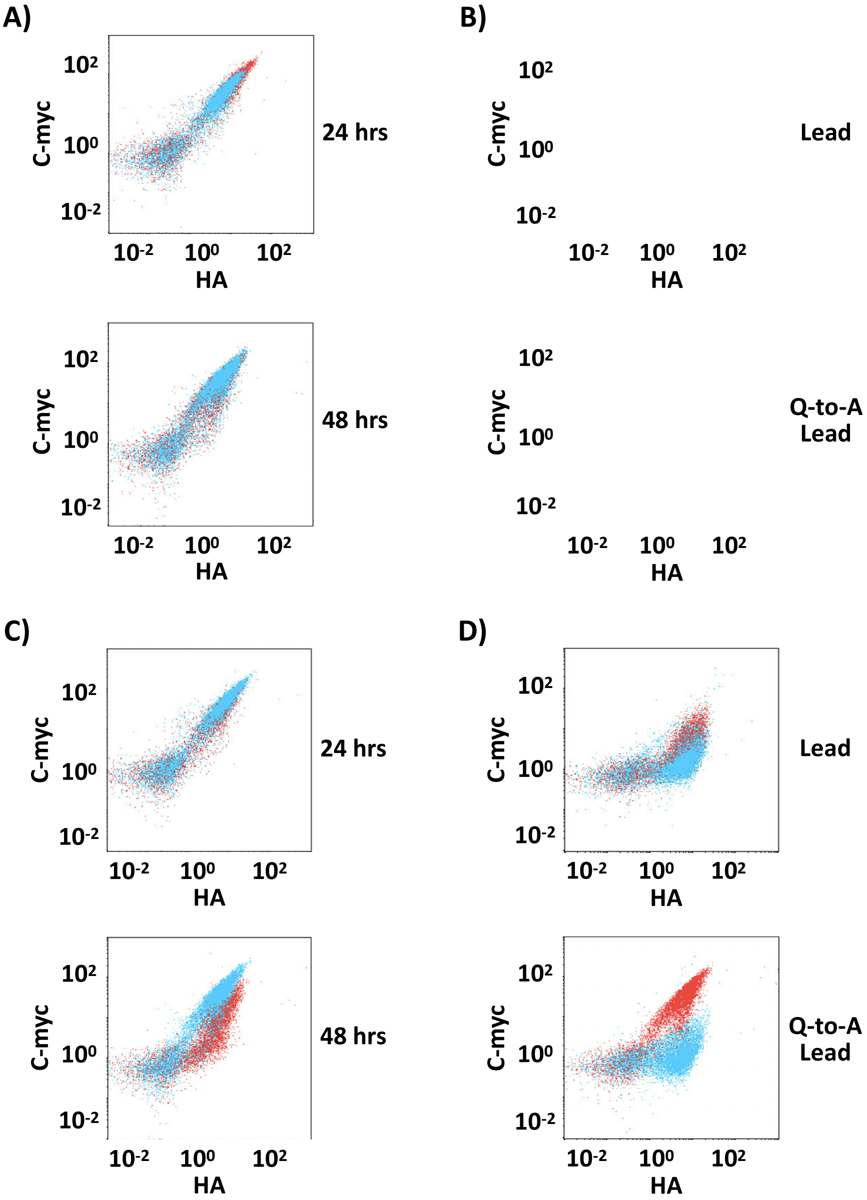

Figure S5. Representative flow cytometry plots comparing the binding of fluorescently labeled antibodies to the HA (x-axis) and c-myc (y-axis) tags displayed on the surface of **(A)** yeast cells engineered with either the "Pro-Kex-TG" vector (red) or the "Pro-Kex-TG w/ Q-to-A SDM" vector (cyan) and induced for either 24 or 48 hrs; **(B)** yeast cells engineered with either the "Pro-Kex-TG" vector or the "Pro-Kex-TG w/ Q-to-A SDM" vector, induced for 48 hrs, and either exposed to TEV protease (cyan) or left untreated (red); **(C)** yeast cells engineered to express either the "active TG, lead/c-myc" construct (red) or the "active TG, Q-to-A lead/c-myc" construct (blue) concurrently with active transglutaminase and induced for either 24 or 48 hrs; and **(D)** yeast cells engineered to express either the "active TG, lead/c-myc" construct (red) or the "active TG, Q-to-A lead/c-myc" construct (blue) concurrently with active transglutaminase, induced for 48 hrs, and either exposed to TEV protease (cyan) or left untreated (red).

S.2.2. Construction and screening of a yeast display library of peptides putatively cyclized by treatment with intracellularly-expressed transglutaminase

Using the system optimized in *Section S.2.1*, we constructed a yeast display library co-expressing randomized linear peptides and intracellular active transglutaminase. Accordingly, the linear hep-

tapeptide library ALQ$X_1X_2X_3X_4X_5X_6X_7$EQKLISEEDL was constructed, wherein the length and design of both leader and c-myc tag segments were maintained as in the optimized construct to ensure alleged cyclization. The library was screened by performing one round of magnetic cell sorting and one round of FACS to isolate binders to N-terminal YAP. While little enrichment was observed after magnetic selection, a marked selection towards YAP binding was observed after FACS (Figure S6).

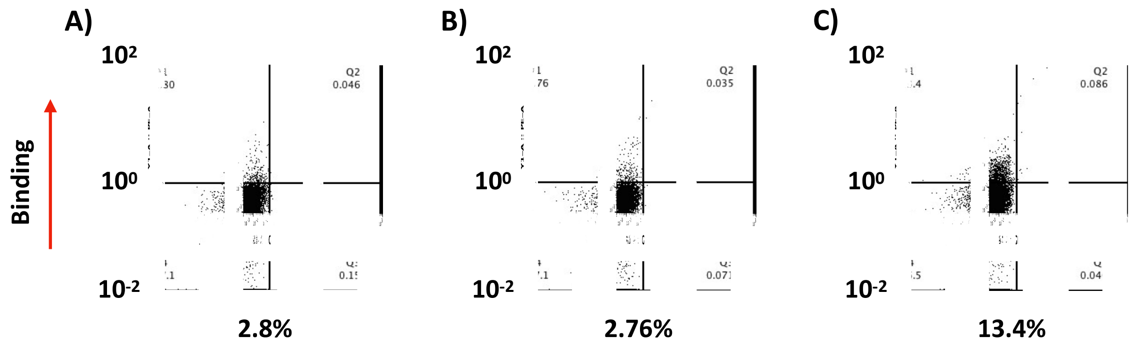

Figure S6. Screening of a yeast display library of peptides putatively cyclized by active transglutaminase expressed intracellularly. The flow cytometry plots detail the binding of N-terminal YAP by cells in **(A)** the naïve library, **(B)** the library after one round of magnetic cell sorting, and **(C)** the library after one round of magnetic cell sorting and one round of FACS. The binding of N-terminal YAP (500 nM) to the cell populations was detected via fluorescent flow cytometry using SA-PE. The fraction (%) of the cell population binding N-terminal YAP *vs.* binding SA-PE only is reported below each plot.

S.2.3. Characterization of N-terminal YAP-binding peptides identified by screening a yeast display library of linear peptides putatively cyclized by intracellular transglutaminase

Of the five clones selected for sequencing, two returned the sequence cyclo[*E*-VQCRGKGEQ-*K*], whereas the other three comprised sequences that were not consistent with the original construct (*i.e.*, lacking the leader sequence or out of frame). The N-terminal YAP:cyclo[*E*-VQCRGKGEQ-*K*] interaction, evaluated via yeast surface titration, returned an apparent K_D of 0.46 ± 0.08 μM. To evaluate the peptide's binding specificity to N-terminal YAP, yeast surface titrations were executed using biotinylated BSA. A comparison of the isotherms generated using N-terminal YAP *vs.* BSA (Figure S7A) indicates that the identified peptide is a selective binder for N-terminal YAP. To assess the binding interaction of linear AVQCRGKGEQK with N-terminal YAP, a similar mutation strategy was carried out to convert the reactive glutamine (Q) residue into an unreactive alanine (A), thus ensuring the surface display of the linear peptide sequence AVQCRGKGEQK. The N-terminal YAP:AVQCRGKGEQK interaction, evaluated via yeast surface titration, returned an apparent K_D of 0.36 ± 0.07 μM (Figure S7B). The apparent similarity in N-terminal YAP-binding strength between the linear and putative cyclic forms of the peptide could be imputed to incomplete modification of the peptide constructs by the intracellular transglutaminase. The screening of yeast cells displaying a combination of cyclic and linear peptides may in fact lead to the identification of peptide sequences that bind the target protein in both configurations and with comparable affinity. While optimization is needed, this proof-of-concept study shows the potential of the proposed method for isolating peptide ligands with selective biorecognition activity from yeast display libraries of peptides putatively cyclized by intracellular transglutaminase. Since TEV treatment is likely to eliminate non-cyclized peptides from the yeast surface (Figure 2), one strategy for selective enrichment of cyclic peptide binders may involve TEV treatment of the library prior to combinatorial screening.

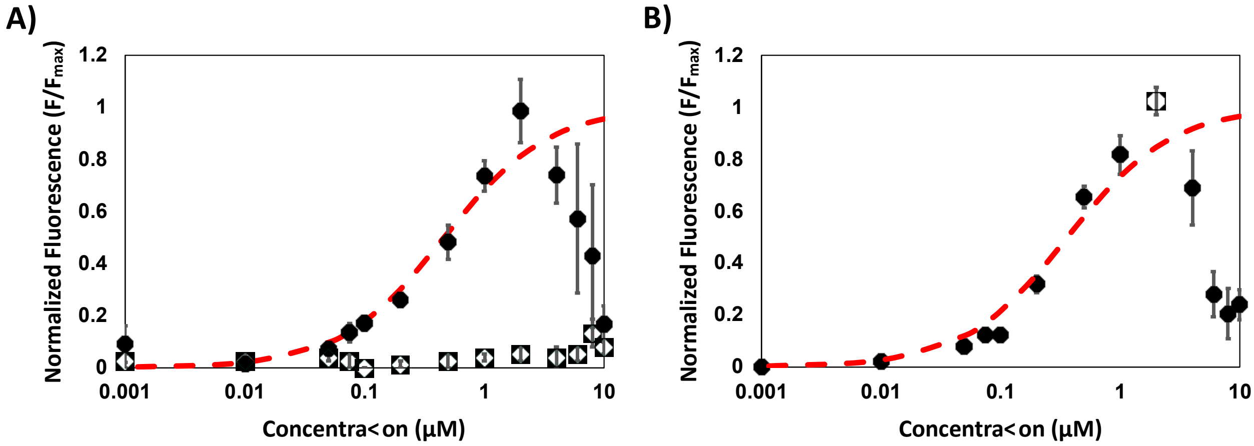

Figure S7. Yeast surface titrations of N-terminal N-YAP (N-YAP, black circles) and BSA (green diamonds) on cells expressing **(A)** cyclo[*E*-VQCRGKGEQ-*K*] and **(B)** its linear counterpart QVQCRGKGEQK. The binding of each biotinylated protein was detected using SA-PE followed by flow cytometry analysis. Data from each independent replicate was normalized to its associated F_{max} value. The values of normalized mean fluorescence intensity for each repeat were fit using a monovalent binding isotherm (red dashed line) to estimate the apparent K_D describing the binding affinity of the yeast-displayed peptides for the soluble protein targets. Error bars correspond to the standard error of the mean from three independent replicates.

Table S1. Oligonucleotide primers.

	Sequence
Pf1	AAAAAAGCTAGCGAGAACCTGTACTTC
Pr1	AAAAAACTCGAGCTATTATAACTCGTC
Pf2	AAAAAACATATGTTCGAAATTCCCGACGATGTG
Pr2	AAAAAACTCGAGCTGATTCATCGCAAAGCGTG
Pf3	AAAAAACATATGATGGATCCTGGGCAGCAG
Pr3	AAAAAACTCGAGCTGTGGCGACTGCGGAGC
Pf4	AAAAAAACCGGTCGCGTTCCTGAAAC
Pr4	AAAAAACTCGAGCTATTAAGCGTAGTC
Pf5	CACCACCACCACGTCGAC
Pr5	TGGAACGTCGTATGGGTAGGA
Pf6	GCGAAAGGTACCCTATTATAATTCATCGT
Pr6	AAAAAAACCGGTGAAGACGAGGACGCA
Pf7	AAAAGGGACTCCGACGACAGAGTA
Pr7	AGGAGCTCTGAAAGAAGG
Pf8	GACTCCGACGACAGAGTAACG
Pr8	GGCTAGCACGCTCGCGAT
Pf9	CCACGCACTGGCATCTGGTTCAAGAGGTG
Pr9	TGGTGGTGGTGGTGCCCT
Pf10	GGTGGAGGCGGTAGCGGA
Pr10	TTTGTTACATCTACACTGTTGTTATCAGAT

Table S2. Gene Blocks.

Gene Block 1	GAGAACCTGTACTTCCAAGGGCACCACCACCACCACCACGCAC-<br>TGCAATCTGGTTCAA-<br>GAGGTGGGGGAGAGCAGAAGCTAATTTCTGAAGAAGACCTGTTT<br>GAACACGACGAGTTATAATAG
Gene Block 2	ATGGATCCTGGGCAGCAGCCGCCACCCCAAC-<br>CAGCGCCTCAAGGTCAGGGGCAAC-<br>CGCCATCACAGCCCCCTCAGGGCCAAGGACCTCCGTCAGGCCCT<br>GGTCAGCCAGCGCCCGCAGCGACACAGGCCG-<br>CACCCCAGGCTCCGCCCGCAGGTCACCAGA-<br>TAGTGCACGTTCGTGGAGACAGTGAAACCGACCTGGAAGCCCTG<br>TTCAATGCGGTTATGAACCCTAAGACCGCTAATGTACCACAAAC-<br>GGTGCCGATGAGACTTA-<br>GAAAACTTCCAGATAGCTTTTTCAAACCACCCGAGCCCAAGTCTC<br>ATAGTCGTCAAGCTTCAACAGACGCAGGAACGGCCGGGGCGTT-<br>GACTCCTCAGCAC-<br>GTGCGGGCTCACTCGTCACCTGCCAGTTTACAGTTGGGTGCTGTCT<br>CACCCGGTACGCTGACCCCTACTGGCGTAG-<br>TGTCCGGTCCGGCCGCAACACCTAC-<br>GGCCCAACACCTTCGTCAATCATCGTTCGAAATTCCCGACGATGT<br>GCCGCTGCCAGCCGGTTGGGA-<br>GATGGCCAAAACTTCCTCAGGCCAGCGTTACTTCTTAAAC-<br>CACATAGATCAGACAACTACCTGGCAGGATCCGCGGAAAGCGAT<br>GTTAAGTCAAATGAATGTGACGGCACCTACATCCCCGCCAG-<br>TCCAGCAGAA-<br>TATGATGAACTCTGCTAGTGGTCCTCTTCCTGATGGGTGGGAACA<br>GGCGATGACGCAAGACGGCGAAATTTATTACATAAACCACAA-<br>GAACAAAACAAC-<br>CTCGTGGCTTGATCCACGGCTGGACCCACGCTTTGCGATGAATCA<br>GCGCATCTCTCAAAGTGCCCCAGTTAAACAACCAC-<br>CGCCCTTAGCTCCGCAG-<br>TCGCCACAGGGTGGGGTGATGGGCGGCAGCAACTCGAATCAGCA<br>GCAGCAAATGCGCTTACAGCAACTTCAGATGGAAAAGGAAC-<br>GCTTACGCTTGAAACAACAA-<br>GAGCTGTTGCGGCAAGAATTAGCATTAAGATCACAGCTGCCTACT<br>TTAGAACAGGACGGGGGTACGCAAAATCCCGTATCCTCGCCGG-<br>GAATGAGTCAGGAGCTTCG-<br>TACTATGACAACGAACTCCTCTGATCCTTTCCTTAACTCCGGGACC<br>TATCACTCGCGCGACGAAAGTACTGATTCCG-<br>GATTGTCCATGTCCTCCTATTCCGTCCCCCG-<br>GACCCCCGACGATTTCCTTAACTCGGTTGACGAAATGGACACTGG<br>GGATACTATAAACCAATCGACCCTGCCTAGCCAGCAAAAC-<br>CGTTTCCCGGATTATTTAGAA-<br>GCTATTCCGGGGACTAACGTAGATTTGGGCACTCTGGAAGGGGAT<br>GGAATGAACATAGAGGGGGAGGAGTTAATGCCGTCTTTGCAA-<br>GAGGCACTGTCTTCTGA-<br>TATCTTAAACGATATGGAAAGCGTGTTGGCCGCCACAAAGTTGGA<br>CAAAGAATCTTTCTTAACATGGTTGTAG
Gene Block 3	ACCGGTCGCGTTCCTGAAACGCAGATGTGCCTCGCGCCGCAC-<br>TGCTCCGAACAATAAAGAT-<br>TCTACAATACTAGCTTTTATGGTTATGAAGAGGAAAAATTGGCAG

	TAACCTGGCCCCACAAACCTTCAAATGAAC-<br>GAATCAAATTAACAACCATAGGATGA-<br>TAATGCGATTAGTTTTTAGCCTTATTTCTGGGGTAATTAATCAGC<br>GAAGCGATGATTTTTGATCTATTAACAGATA-<br>TATAAATGCAAAAACTGCATAACCAC-<br>TTTAACTAATACTTTCAACATTTTCGGTTTGTATTACTTCTTATTCA<br>AATGTAATAAAAGTATCAACAAAAAATTGTTAATATACCTC-<br>TATACTTTAACGTCAAGGA-<br>GAAAAAACCCCGGATCGAATTCCCTACTTCATACATTTTCAATTA<br>AGATGCAGTTACTTCGCTGTTTTTCAA-<br>TATTTTCTGTTATTGCTTCAGTTTTAGCACAG-<br>GAACTGACAACTATATGCGAGCAAATCCCCTCACCAACTTTAGA<br>ATCGACGCCGTACTCTTTGTCAACGACTACTATTTTGGCCAACGG-<br>GAAGGCAATGCAAGGAG-<br>TTTTTGAATATTACAAATCAGTAACGTTTGTCAGTAATTGCGGTTC<br>TCACCCCTCAACAACTAGCAAAGGCAGCCCCATAAACACACAG-<br>TATGTTTTTGGTGGAG-<br>GAGGCTCTGGTGGAGGCGGTAGCGGAGGCGGAGGGTCGGAGAA<br>CCTGTACTTCCAAGGGCATCATCACCACCACCAC-<br>GTCGACTGGGCGCTACAACGTCCG-<br>CATGGGGGAGGAAAGTCCGGATCCTACCCATACGACGTTCCAGA<br>CTACGCTTAATAG
Gene Block 4	GGTACCCTATTATAATTCATCGTGTTCAAAC-<br>CATGGCGCCAACCCGGCTTTTCCTT-<br>GTCTGGGTCTATGTTCTAGCATACGTGGGGAGGGGGAGGGACTGA<br>CCACGTAAGGTTCCCCACGGTCAAAATCACTG-<br>TAACCCTCAGATCCAGTAGCAGAG-<br>GATGCTCTCGTCAAACATGCATGGCAGCCCAGACTGCCGTTCGGG<br>GCATGATAGTGATTTCCGTGGGTCCAAACTGTCTTGTCTG-<br>CATCGGCTTCTGTTT-<br>GTGCGCCGAACCAACCATAGTCAAAGTTGACAAAACCTTCTCCC<br>GGAGAGGTCGGGGACCTTGGGATATTTCTATCTCTT-<br>GACATGTCAAC-<br>CAGACCCGTCCCTGGCGCACTACGAAAAGCATCGGGGTCCCCGT<br>ATTTACGCTTATCCGCGGAGGAGGATCTGTCCTGGCCAC-<br>TCCAAAAATGCTTACTATAAA-<br>TAACAGCCTTCATTCTAGATGGATCATGGTTACCTCCGTTACGCTC<br>CTTGAAGCTTGGTGTGTTTCTCAAGGCGGAATAGAAC-<br>GGACTTCTCGCGTCCTCGTTAC-<br>GCAGTGCGTCATTACCGTTTGCAAGTTCCTTTTTAAGGTTGTCCAG<br>GTAGGCTGATTCGTCGTGAGCATTTTCTAAA-<br>GCTCTGTTCATAACACTGGCGACTTCCCTT-<br>GCCCTCTGAAAGCCCTTCTCTTCGTCGAATGACTCCTTGGCAACA<br>CGTCCCTCAAATTCGGCACGAGTCTCGCCG-<br>GATCTGGGCCTGCCGTTCTTTAGCTCATTCTTAAACCTGTCCTCGT<br>CGAAAGAAGCGAACGCTAGACGATTGGTAGGATATTGCCCTGAG-<br>TTAACCCAAGTAACTCC-<br>TACGCACCCGTAAGAAAGCCATTCCCTCTGCTCCTCGGTCATCTG<br>CTGTTTCCTGCCGTCCCTATGGGAATAAACTTGTTGCCAC-<br>TTCCTAATATAATTATTCAC-<br>GACGGTTTCTGCCCTTCCATAGCTTGGACGATAGGGATCAGGCAT

	CCTATCCAAGGGCTCTGCAGGGGGCGTTACTCTGTCGTCGGAG-<br>TCAGGAGCTCTGAAA-<br>GAAGGGCCGGCACTTGACGCCGCCGGAGCAGACTCATTCAGCGC<br>GTTGATATTTGCGACGTCATCAGCCGTCAATCTG-<br>TATGTCTCCGCGTATGATTTT-<br>GTTTCCTTTCCACGGGCCATCGTTGGCGGGGGCCTGGCTTGTCCAA<br>CAGCGTGAATCCTCCTATGCATGGCTAGCACGCTCGCGATTAC-<br>GGAGAAGATACTA-<br>AAACAACGTAGAAGCTGCATCCTAGGAATTCCTTGTTGAATTCGA<br>ATTTTCAAAAATTCTTACTTTTTTTTTGGATGGACGCAAA-<br>GAAGTTTAA-<br>TAATCATATTACATGGCATTACCACCATATACATATCCATATACA<br>TATCCATATCTAATCTTACTTATATGTTGTGGAAATGTAAA-<br>GAGCCCCATTATCTTAGCCTAAAAAAACCTTCTCTTTGGAACTTTC<br>AGTAATACGCTTAACTGCTCATTGCTATATTGAAGTAC-<br>GGATTAGAA-<br>GCCGCCGAGCGGGTGACAGCCCTCCGAAGGAAGACTCTCCTCCG<br>TGCGTCCTCGTCTTCACCGGT

Table S3. Oligonucleotides encoding randomized peptides.

	**Sequence**
Oligo 1	CACCACCACCACGTCGACTGGGCGCTACAANNKNNKNNKNNKNN-<br>KNNKNNKNNKAAGTCCGGATCCTACCCATACGACGTTCCA
Oligo 2	GGTGGAGGCGGTAGCGGAGGCGGAGGGTCGGCTAGCGAGAACCTG-<br>TACTTCCAAGGGCAC-<br>CACCACCACCACCACGCACTGCAANNKNNKNNKNNKNNKNNKNNK<br>GAGCAGAAGCTAATTTCTGAAGAAGACCTGTTTGAACACGACGAG-<br>TTATAATAGCTCGAGATCTGATAACAACAGTGTAGATGTAACAAA
